# Supplementary material for: Evaluation of postural therapy using lateral position according to fetal back orientation on breech presentation and breech recurrence (BRLT study): An open-label randomized controlled trial
Source: PLoS Med. 2025 Mar 25;22(3):e1004555. doi: 10.1371/journal.pmed.1004555 (PMC11936219; doi:10.1371/journal.pmed.1004555)
Supplement: S1 Table — (DOCX) [file pmed.1004555.s001.docx]

**Supporting Information (Supplemental Table)**

**Table S. Primary and Secondary Outcomes in Primiparous and Multiparous Women.**
