# Supplementary material for: Evaluation of postural therapy using lateral position according to fetal back orientation on breech presentation and breech recurrence (BRLT study): An open-label randomized controlled trial
Source: PLoS Med. 2025 Mar 25;22(3):e1004555. doi: 10.1371/journal.pmed.1004555 (PMC11936219; doi:10.1371/journal.pmed.1004555)
Supplement: S2 Protocol — (DOCX) [file pmed.1004555.s003.docx]

**Study Protocol for Medical Research Involving Human Subjects**

**1. Title^※^**

**A randomized controlled trial of cephalic version for breech presentation in the third trimester by lateral postural management without knee-chest position (BRLT study)**

Version：2.1

Date：6/9/2022

※This protocol was made to comply with the SPIRIT 2013 Statement, which provides recommendations for standard protocol items in intervention trials; the corresponding item numbers in the SPIRIT 2013 checklist are appended after the title of each section by (S item number)

**2. Research Implementation Structure（S5a）**

①Institution (Department)

Nippon Medical School Musashikosugi Hospital Obstetrics and Gynecology

②Name, Affiliation, Job title, Role

1) principal investigator

Name　　　　Hiroki Shinmura

Affiliation, Job title: Obstetrics and Gynecology Assistant Professor

Role: conception, formulation of protocol, data management, data evaluation

2) member of the research project

Name　　　　Shunji Suzuki

Affiliation, Job title: Obstetrics and Gynecology　Professors

Role: formulation of protocol, data analysis and evaluation

Name　　　　Takashi Matsushima

Affiliation, Job title: Obstetrics and Gynecology Associate Professors

Role: formulation of protocol, data analysis and evaluation

Name　　　Honglian Shi

Affiliation, Job title: Obstetrics and　Gynecology　Resident

Role: formulation of protocol, data collection, data evaluation

Name　　　　Ayako Takizawa

Affiliation, Job title: Obstetrics and Gynecology Assistant Professor

Role: formulation of protocol, data collection, data evaluation

Name　　　　Asako Watanabe

Affiliation, Job title: Obstetrics and Gynecology Assistant Professor

Role: formulation of protocol, data collection, data evaluation

Name　　　　Eika Harigane

Affiliation, Job title: Obstetrics and Gynecology Assistant Professor

Role: formulation of protocol, data collection, data evaluation

Name　　　　Asako Nagashima

Affiliation, Job title: Obstetrics and Gynecology Assistant Professor

Role: formulation of protocol, data collection, data evaluation

Name　　　　Mayu Yamada

　Affiliation, Job title: Obstetrics and Gynecology　Time-saving Assistant Professor

Role: formulation of protocol, data collection, data evaluation

Name　　　　Youhei Tsunoda

Affiliation, Job title: Obstetrics and Gynecology Assistant Professor

Role: formulation of protocol, data collection, data evaluation

Name　　　　Ryuhei Kurashina

Affiliation, Job title: Obstetrics and Gynecology　Lecturer

Role: formulation of protocol, data collection, data evaluation

Name　　　　Go Ichikawa

Affiliation, Job title: Obstetrics and Gynecology Assistant Professor

Role: formulation of protocol, data collection, data evaluation

3) Personal Information Administrator (essential for human genome and genetic analysis research)

Name　　　Hiroki Shinmura

Affiliation, Job title: Obstetrics and Gynecology Assistant Professor

1. In case of multicenter studies

Not applicable.

**３．Purpose and Significance of the Study**

1. **Background of the Study（S6a、6b）**

　Since the U.S. guidelines recommended abdominal cesarean section for breech delivery, the number of facilities performing cesarean sections has increased in Japan.^2)^ At present, most cases of pelvic position are delivered by cesarean section at our hospital. External rotation is expected to be effective in correcting the breech presentaionn and is recommended in the guidelines of developed countries (the United States, the United Kingdom, the Netherlands, and France), but it is an invasive procedure.^3-6)^

Other known methods of correction for breech fetus include the knee-chest position (so-called breech exercise in Japan, Figure 1), the supine position with pelvic elevation (Indian version, Figure 2), and the lateral position (Figure 3), and acupuncture and moxibustion, although the effectiveness of both methods is controversial.^7-12)^ A 2012 Cochrane Library review of well-controlled trials concluded that there is insufficient evidence to support the use of postural therapy for the pelvic position.^7)^ The review included six randomized controlled trials, four of which evaluated the knee-chest position and two of which evaluated the supine position with pelvic elevation (Indian version).^13-18)^ Because pregnant women may complain of breathlessness and uterine contractions, and because of the lack of evidence for the knee-chest position, some centers in Japan do not recommend this technique at present.


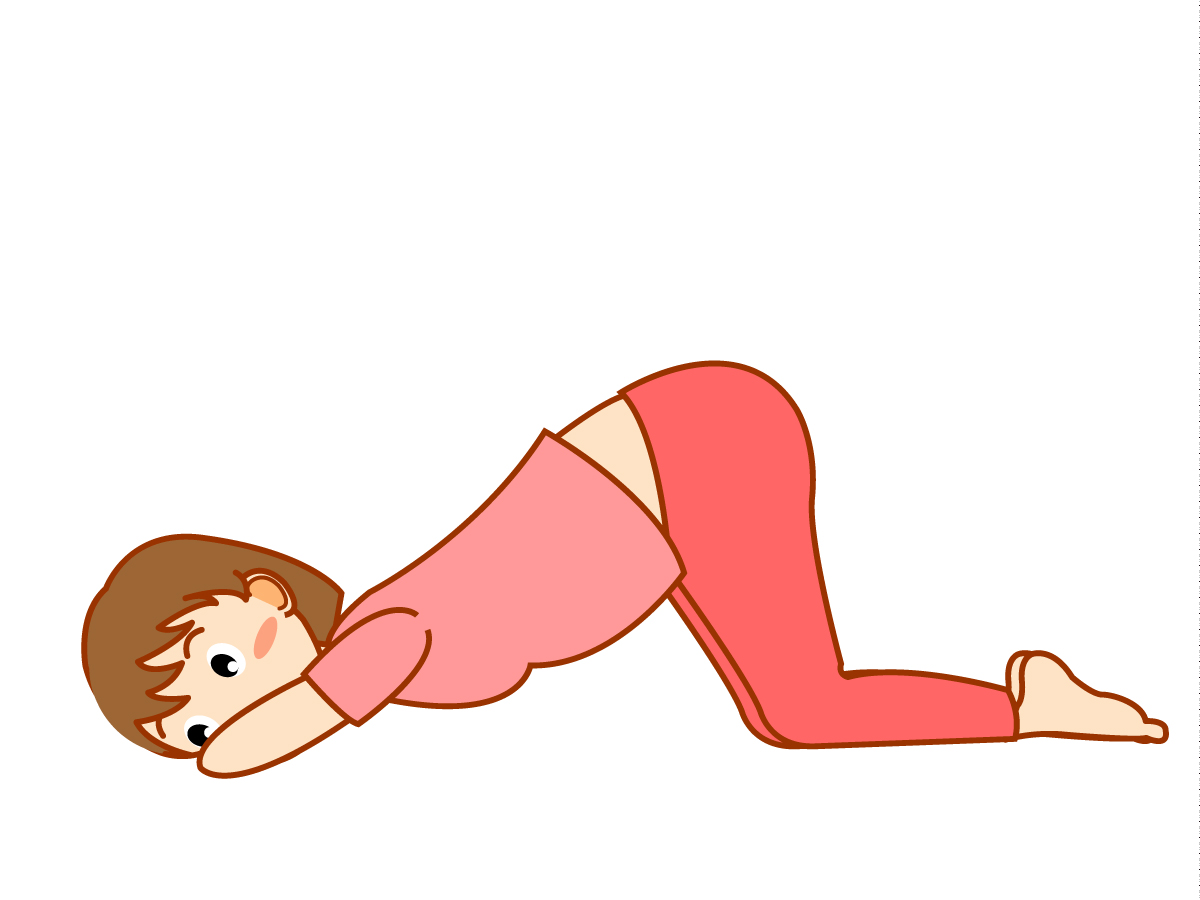


Figure 1　Knee-chest position


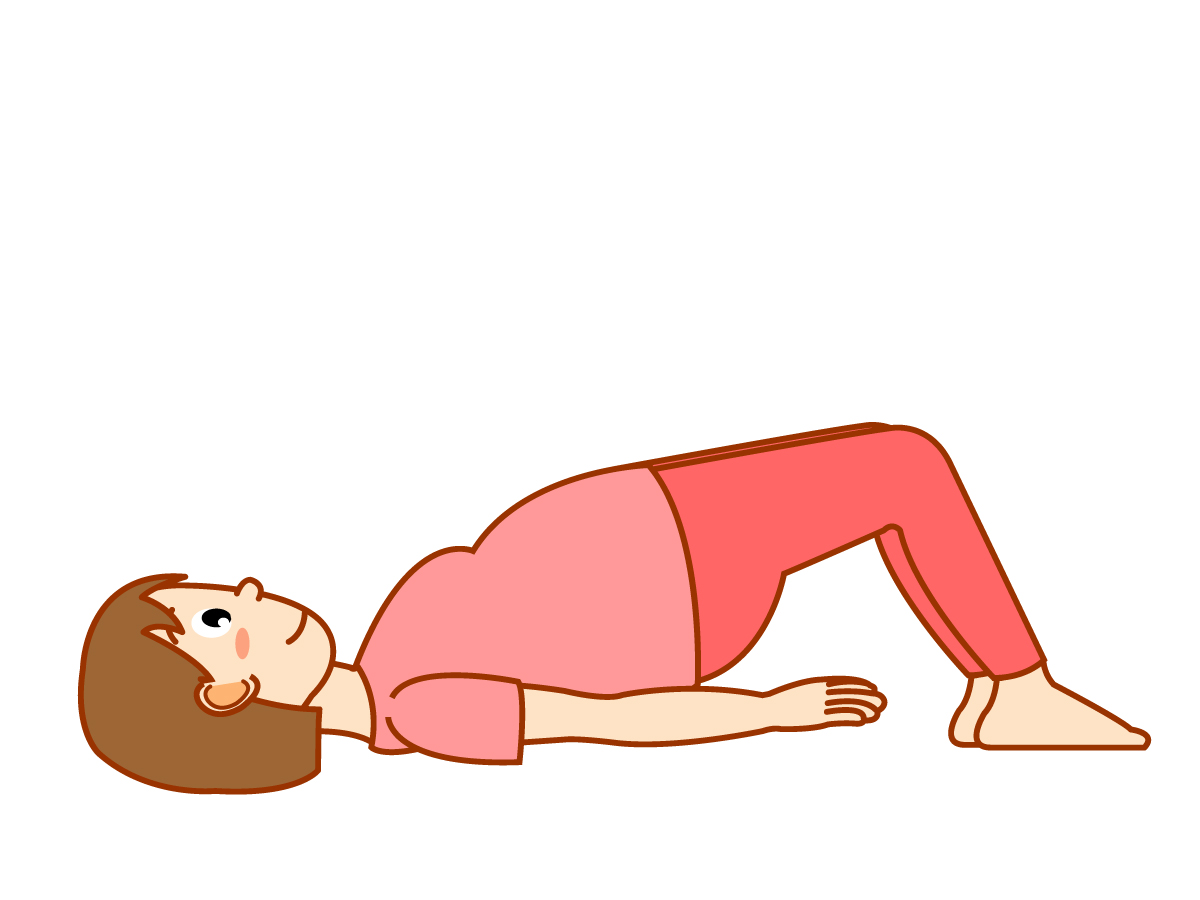


Figure 2　Indian version


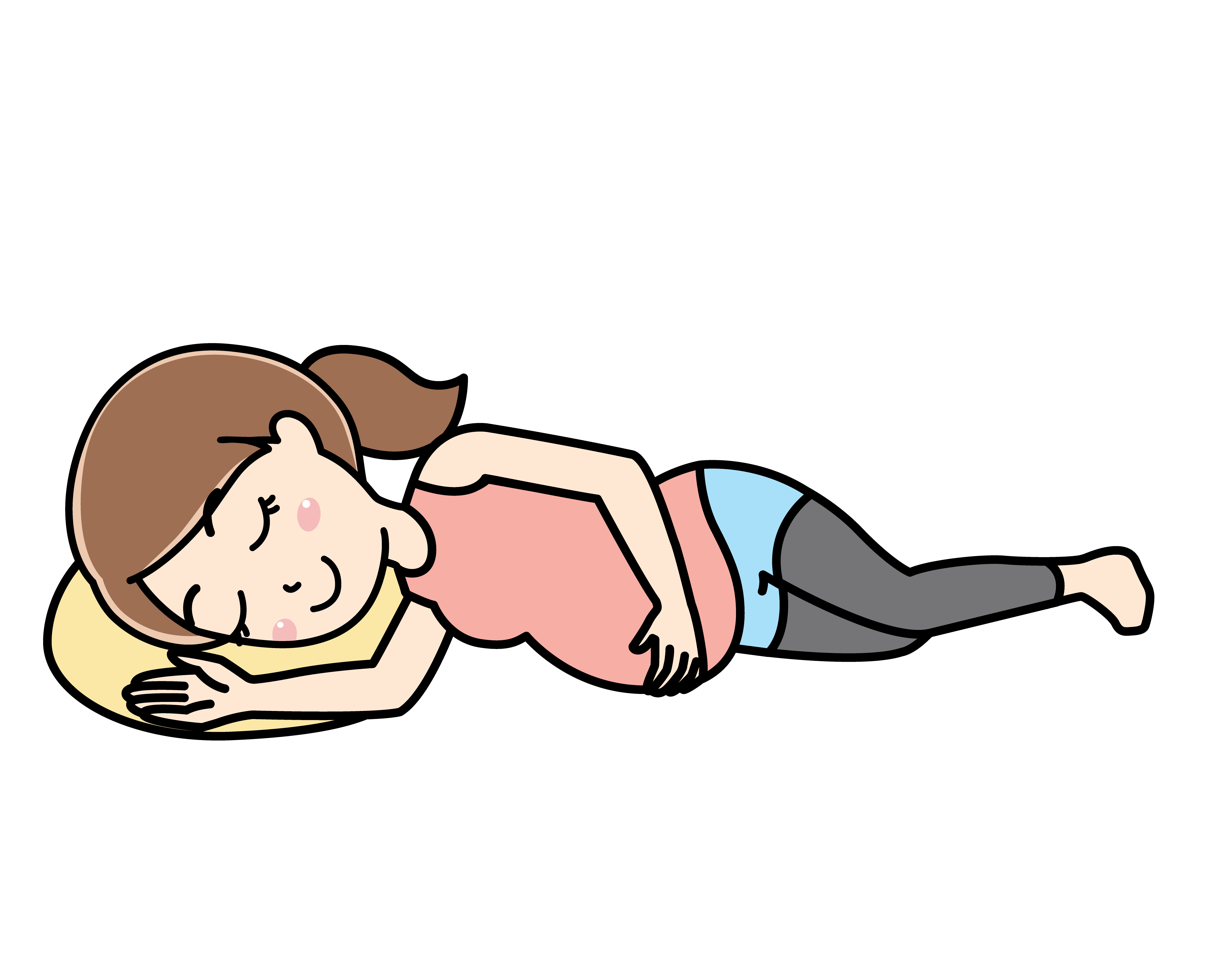


Figure 3　Lateral position

Although there is little evidence for the lateral position method (sleeping on the right side if the baby's back is on the mother's left in the first orientation and sleeping on the left side if the baby's back is on the mother's right in the second orientation) and it was not included in the evaluation of the Cochrane review mentioned above, it has been practiced in obstetrics since it was proposed by Taoka in 1943 and is still used today by obstetricians who have experienced its effects on the breech presentation in their clinical practice.^19,20)^ The lateral recumbent position is also the first position to be taught to pregnant women during frequent uterine contractions, position-induced mood discomfort (e.g., supine hypotension syndrome), or fetal dysfunction, and is safer, simpler than the knee-chest position, and cost-free. According to a retrospective cohort study at our hospital, the probability of returning to the cephalic position after 2 weeks was significantly higher in the group that was instructed only in the lateral position without the knee-chest method compared to the group that was not instructed (81.8 % (18/22), vs. 57.6 % (34/59), p = 0.043). If a simple, minimally invasive, and cost-free lateral positioning intervention for pelvic position in late pregnancy can correct the pelvic position, high-risk deliveries due to pelvic position may be reduced.

Here we formulated the following hypothesis. The chest-knee position is a non-physiological position that stimulates uterine contractions and prevents fetal self-rotation. In the lateral position, the uterus is at rest, so uterine contractions are less likely to be evoked, and the gravity of lying on the opposite side of the baby's back encourages fetal self-rotation. Conversely, lying with the dorsal side down stabilizes the fetal position." Applying this hypothesis to the correction of the breech presentation, it is expected that a combination of postural therapy that does not involve the knee-chest positioning for the pelvic position, but rather the lateral position and the reverse lateral position after cephalic version, would be most effective. For the sake of convenience, this combination of these postural therapies is collectively referred to as the “Japanese Medical School style postural therapy”. Since the peak amniotic fluid volume is generally around 32 weeks gestation,^21)^ starting the lateral position by then may promote fetal self-rotation, and since previous studies have found no problems with the lateral position. Therefore, starting the intervention from 28+0 weeks to 30+0 weeks may increase the effectiveness of continuous lateral position instruction.

1. **Objectives of the Study（S7）**

　To evaluate the usefulness of the Japanese Medical School style postural therapy for breech presentation in late pregnancy (knee-chest position is not performed, but the lateral position method and the reverse lateral position after head version are performed).

1. **Significance of the Study**

Cephalic version is clinically important to avoid cesarean section. It is known that the pelvic position is often corrected spontaneously in the early weeks, but the rate of spontaneous version declines as the weeks progress.^21)^ Most facilities perform external rotation around 35 to 37 weeks, but there is no established method for treatment from 28 to 36 weeks, when the rate of spontaneous correction declines. The lateral position can be easily taught during outpatient antenatal checkups. It is also generally the first position to be taught to pregnant women when they are uncomfortable, and is less likely to cause discomfort than the knee-chest position, making it safe and cost-free. If the effectiveness of the lateral position is demonstrated, minimally invasive and cost-free treatment of the pelvic position can be performed after 28 weeks, potentially reducing the number of high-risk deliveries due to the breech presentation.

**４． Methods and duration of the study**

1. **Type and design of research（S8）**

　This is an open-label, parallel, randomized, controlled trial of the superiority of the NMS-style postural therapy. The allocation ratio is intervention group　vs. control group = 1:1.

1. **Methods of Research（S13）**

a. Recruitment of Study Participants

　Women aged 20 years or older (due to the social reason that under 20 years of age, the consent of a parent or guardian is required and may not be obtained at the time of enrollment) who underwent antenatal care at Nippon Medical School Musashikosugi Hospital between 28 weeks 0 days and 30 weeks 0 days gestation between April 1, 2021 and March 31, 2024, and who had a pelvic position on screening ultrasound examination, will be included. Exclusion criteria were as follows: already receiving treatment for pelvic position, placental malposition, previous cesarean section, scheduled cesarean section after uterine myoma surgery, etc., scheduled delivery at another hospital, multiple pregnancies, transverse position, and complications considered risky for performing positional therapy (such as history of heart disease). If determined to be eligible, they would be invited to participate in the study and consent would be obtained at that day. To avoid variation among obstetricians in charge of recruitment, the explanation of participation in the study will use standardized content as shown in the "Sample Explanation to Research Participants" in the Appendix.

b. Allocation of research subjects（**S16a、16b、16c、17a、17b**）

Once the consent has been obtained, apart from the obstetrician who has recruited the participants, the outpatient staff in charge of antenatal checkups will randomly assign the participant to the two groups using the allocation table provided in the antenatal checkup booth.

The allocation table is computer-generated using a combination of stratified randomization and random block size methods, and is prepared by the independent investigator according to the "Allocation Procedures" in the attached document. Because the previous childbirth history has a significant impact on cephalic version, previous studies on other postural therapies have found that the variation in previous childbirth history in both groups of randomized controlled trials is an important factor in assessing the quality of the study. Therefore, in this study, stratified randomization will be used for primiparous mothers and multiparous mothers in order to control for the proportion of primiparous and multiparous mothers. The allocation table is numbered sequentially, but the allocation group is concealed by a concealment sticker, so that neither the enrollee nor the allocator knows the allocation group until the concealment sticker is removed after consent is obtained. Once the allocation group is determined, the outpatient staff informs the obstetrician, except for the statistician, of the allocation group, and the obstetrician reports to the study participants on the spot whether they have been assigned to the intervention or control group.

c. Intervention（**S11a、11b、11c、11d**）

The intervention group will be instructed in the lateral position on site by the physician in charge of antenatal checkups. They are instructed to lie on their right side for the first breech presentation (the fetal back is to the mother's left side) and on their left side for the second breech presentation (the fetal back is to the mother's right side). The participant should be encouraged to lie on her side three times a day for 15 minutes at a time, especially at the beginning of sleep. Other treatment for breech presentation, such as knee-chest position (so-called pelvic position exercise), should be avoided. If discomfort occurs, the patient should be instructed to stop lying on her side and change positions immediately, and be asked to report events. If the patient does not feel uncomfortable, she will be allowed to perform the postures for more than 15 minutes. For example, if the patient can perform the indicated postures for more than 45 minutes, she will be considered to have performed the postures at least three times and the posture therapy for that day is considered to have been successful. The patients are asked to write down the positions they actually took on the attached "Position Recording Form," and the form is collected at the next antenatal checkup. From the collected record forms, the implementation rate of the taught positions is calculated, and a successful implementation is defined as greater than 50% of successful days in a 2-week period. In the intervention group, the fetal position (pelvic, head, transverse, etc.) and fetal orientation (the direction of the fetal back, with the back of the fetus on the mother's left in the first fetal position and on the mother's right in the second fetal position) are confirmed by ultrasound tomography at every 2-week checkup, and the lateral position method described above is instructed if the fetus is in pelvic position. If the patient is placed in the transverse position during the process, the patient should be instructed to lie on the right side if the infant's head is on the left side of the mother, and to lie on the left side if the infant's head is on the right side of the mother. From the point of change to the cephalic position, the participant should be instructed in the reverse lateral position, in the opposite direction from the lateral position. The first head position (with the fetal back to the mother's left side) is taught in the left side lying position, and the second head position (with the fetal back to the mother's right side) is taught in the right lying position. In the case of an oblique position, the fetal position is considered to be the same as when the infant's head is lifted toward the mother's head. If the infant's back is centered, the orientation of the fetus should be determined based on the orientation of the infant's femur. If it is difficult to determine the orientation, the position of the fetal back should be determined based on the position of the fetal vertebrae in relation to the maternal midline. In previous studies, there were no cases in which fetal orientation could not be determined. Instruction will be continued until delivery.

The control group will not receive specific positional guidance and will continue to receive antenatal care in accordance with the Obstetrics and Gynecology Clinical Practice Guidelines, Obstetrics Edition 2020. The patient is asked to fill out the positions actually taken on the attached document, "Position Recording Form," and the form is collected at the next antenatal checkup.

Participants who are in the non-cephalic position at 37 weeks gestation and who wish to undergo external rotation (a procedure in which the fetus is manually rotated from the pelvic position to the head-first position in the uterus from the surface of the body) or vaginal delivery will be referred to a facility that offers external rotation or breech presentation vaginal delivery. If neither external rotation nor vaginal delivery is desired and the non-cephalic position has not improved after 37 weeks, the patient is delivered by scheduled cesarean section at 38 weeks, as has been our policy. Cases of cephalic position are treated in the same manner as normal pregnant women.

d. Data collection（**S18a、18b、22**）

Participants in both groups will have their fetal position and orientation confirmed by ultrasound tomography at each checkup and recorded in the medical record. Other items to be recorded are age, height, weight, fetal position and orientation, type of breech presentation (complete, incomplete, transverse, foot), amniotic fluid volume, placental position (fundus, anterior wall, posterior wall, right lateral wall, left lateral wall), mode of delivery, birth weight, umbilical cord artery pH at birth, Apgar score 1 min/5 min, complications (gestational hypertension, gestational diabetes, intrauterine fetal growth retardation, impending preterm delivery, uterine malformation, abnormal amniotic fluid volume, fetal malformation, etc.), and adverse events due to positional guidance. In order to prevent dropout from the study, we will prepare standardized answers as shown in the "Sample Explanation to Study Participants" in the attached document to prevent differences in responses among obstetricians in charge of antenatal checkups to questions that may be expected from study participants. To prevent missing values, meetings should be held regularly among researchers to confirm eligibility and exclusion criteria as well as to explain the progress of the study and to share information about data that are likely to be missing.

e. Outcome（**S12**）

The primary endpoint will be the rate of head conversion at 37 weeks gestation. Secondary endpoints are cesarean delivery rate, cesarean delivery rate with breech presentation as an indication, head position conversion rate after 2, 4, and 6 weeks of instruction, breech presentation recurrence rate after cephalic version, and occurrence of adverse events associated with the instruction. Evaluation will be done by comparing the percentage of cephalic fetuses in both groups.

f. Statistical Analysis（**S20a、20b、20c**）

　Intention-to-treat analysis will be performed as the main result. Per-protocol analyses will also be performed to evaluate the effecacy of the pure lateral position method, excluding participants in the control group with a theoretically correct lateral posture attempt rate greater than 50% and those in the intervention group with a lateral position successfull rate of less than 50%. Outcomes are categorical variables and will be evaluated using the chi-square test or Fisher's exact probability test. Relative risk (RR), relative risk reduction (RRR), and absolute risk reduction (ARR) will be determined with 95% confidence intervals. The chi-square test or Fisher's exact probability test is used to evaluate qualitative variables, and the t-test or Mann-Whitney U test is used to evaluate quantitative variables if they are normally distributed or not normally distributed. The significance level is set at p < 0.05 for all tests. Statistical analyses will be performed using the Statistical Package for the Social Sciences software for Windows (version 26.0, IBM Corp., Armonk, NY, the USA). Subgroup analyses will be limited to multiparous and first-time mothers. Missing values will be reported each time they are used, using the pairwise deletion method for categorical variables and the mean assignment method for continuous variables. The detailed statistical analysis plan for these and other exploratory analyses will be formulated. The statistical analysis plan may be revised as appropriate depending on the distribution of data and missing values.

g. Number of cases required（**S14**）

The number of samples needed to achieve the study objectives was determined by a preliminary power analysis. α error was set at 0.05, following convention. Power (1-β) was set at 0.80, following customary practice. Based on a previous study at our hospital, we set the rate of head position at delivery to 94% in the group instructed in the JMHJ style of postural therapy. As for the control group, the rate of spontaneous correction from the breech presentation at 28 weeks to the head position at delivery was 75%-85% according to reports, and the head position rate at delivery was 77% in the previous study at our hospital, we set the head position rate at delivery for the control group at 80%.^23-26)^ The effect size was φ = 0.2081454 for the 94% of intervention group versus 80% of control group, and G*Power 3.1 (version 3.1, Faul, Erdfelder, Lang, and Buchner, Düsseldorf, Germany) was used to obtain the total number of cases n = 182 required. Considering that there would be several dropouts from the study (within 10%), the target sample size was set at 200 for both groups combined (180 before the protocol change).

h. Research Ethics Approval（**S24、25**）

This protocol was prepared in accordance with the Declaration of Helsinki and the Ethical Guidelines for Life Sciences and Medical Research Involving Human Subjects and the CONSORT 2013 Declaration. This protocol was reviewed and approved by the Nippon Medical School Musashikosugi Hospital Ethics Review Board (approved March 9, 2021, receipt number 599-2-64). In the future, the Ethics Review Board will approve any important changes in the implementation plan (e.g., outcomes, number of participants to be recruited, duration, research methods, etc.) in a timely manner.

i. Research Conceptual Diagram


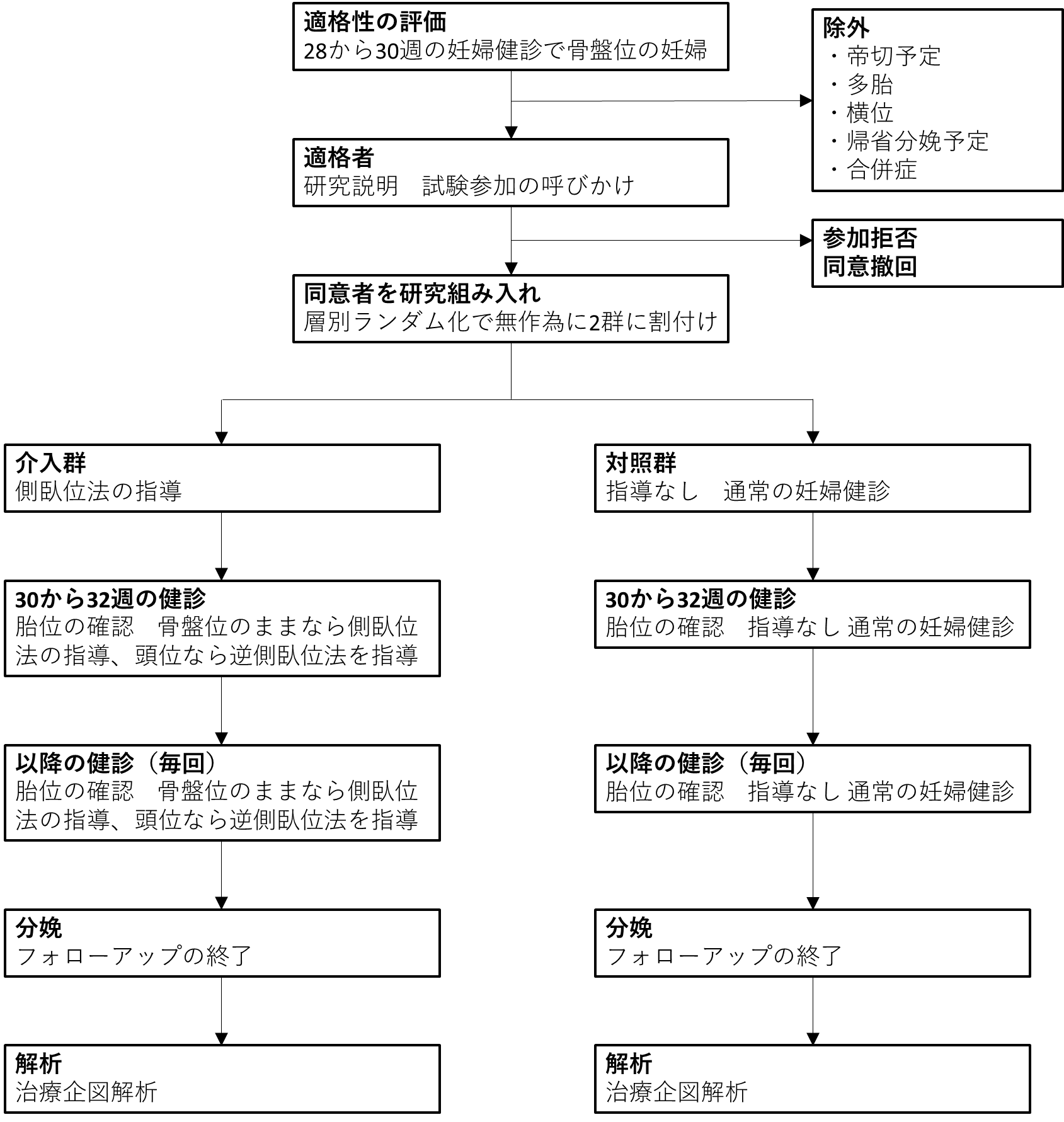


j. Assessment Schedule

1. **Research Period**

Planned Research Period：

April 1, 2021 - March 31, 2024 (3 years and 0 months)

The duration of the study was established based on the target sample size and the number of eligible participants per year in prior studies at our hospital.

　Recruitment of participants will be terminated when the trial reaches the target sample size of 200 participants.

**５．Selection policy for participants（S9、10）**

　Women aged 20 years or older who underwent a prenatal checkup at Nippon Medical School Musashikosugi Hospital between April 1, 2021 and March 31, 2024 at 28 weeks 0 days to 30 weeks 0 days gestation and whose ultrasound tomography scan showed a pelvic position are eligible for the trial.

Exclusion criteria are as follows:

・**Patients who have undergone treatment for breech presentation prior to study entry.**

**・Placenta previa, previous cesarean section, or cesarean section scheduled after uterine myomas surgery**

**・Scheduled delivery at another hospital**

**・Multiple pregnancies**

**・Transverse position**

**・Complications that may pose a risk to the use of postural therapy (e.g., history of cardiac disease)**

**６．Procedures for obtaining informed consent (IC)（S15、26a、26b）**

Explain the study and obtain IC when the breech presentation is recognized on ultrasound tomography during the antenatal checkup and the patient becomes a potential participant. Explain at the same time that consent can be withdrawn at any time. The instructions and consent form to be used regarding the content of the explanation and consent should be attached. We will invite all eligible pregnant women to participate in the study in order to incorporate enough participants to reach the target sample size. To prevent protocol deviations in the control group, explain that postural therapy and acupuncture are not recommended due to insufficient evidence, and that the literature shows that even in the pelvic position at the time of inclusion, there is approximately an 80% chance of returning to the head-first position.

**①　When receiving IC from a surrogate, etc., the procedure**

Not applicable.

**②　If informed assent is obtained, the procedure (only if IC is obtained from a surrogate or others)**

Not applicable.

**７． Handling of Personal Information（S19、27、29）**

　Personal information is anonymized by eliminating it when information is examined and recorded from the medical record. Personal identification codes will not be recorded. Instead of medical record numbers, numbers for research and study will be assigned in sequence and managed. No correspondence table will be created. In principle, only the person in charge handles the data, and an agreement is made that other researchers will not have access to the analyzed data. The principal investigator refers only to the results after data analysis.

**８．The burdens and anticipated risks and benefits to the research participants, a comprehensive assessment of these burdens and risks, and measures to minimize such burdens and risks**

　No burden to the study participants will occur. The intervention group may correct the breech presentation. In the NMS style of postural therapy, instruction in positions that may cause health hazards will not be included. There are no reports of adverse health effects due to the positions taught, either in actual clinical practice or in a literature search. The lateral position is also the first position to be taught to pregnant women during frequent uterine contractions and position-induced discomfort (e.g., supine hypotension), and the potential for adverse health effects from this study, including minor symptoms, is extremely low. However, in the event of adverse events, such as discomfort, the patient should be instructed to immediately discontinue the lateral position and change posture. Participants are instructed to report any adverse events to the person in charge at the next checkup. In the control group, no disadvantage will be incurred because they will only receive a normal antenatal checkup in Japan. If the results of this study demonstrate the effectiveness of the NMS-style postural therapy, it may be possible to avoid a breech presentation delivery of the study participants.

**９．Storage and disposal of samples and information (including materials pertaining to information used in research) Methods of storage and disposal of（S19、27）**

The database obtained from the study will be kept in the Department for 5 years after the completion of the study or 3 years after the publication of the results. Data that are no longer needed will be deleted or disposed of as appropriate.

**１０．Details and Method of Reporting to the responsible person of the Research Institution（S23）**

　At least once a year, a progress report (annual report) on the research will be made to the responsible person of the research institution. If a serious adverse event occurs or information that compromises the validity of the research is obtained, the principal investigator will promptly report this to the responsible person of the research institution. Reports of discontinuation or termination of the research will also be made on a case-by-case basis.

**１１．Sources of funding for the research, etc., conflicts of interest related to the research by the research institution, and conflicts of interest related to the research by the researcher, etc., including personal earnings（S4、5b、5c、5d、19、28）**

The Investigators have no conflicts of interest to disclose with respect to this study. A separate declaration of conflicts of interest should be submitted. This study is not funded by any organization. For this study, a personal computer owned by the Department of Women's Health and Obstetrics will be used for data collection and analysis. The statistical software is also owned by the medical office. The printing of documents and analysis data will be done using a printer owned by the medical office. Other miscellaneous expenses will be funded by the medical office. No revenue is expected from this study.

There is no possibility of mixed treatment in this study.

**１２．Methods of disclosing information on research（S2a、2b、31a、31b、31c）**

Since this is an intervention study, we plan to register the study in the following database, one of the national clinical research databases designated by the World Health Organization (WHO Primary Registry), and make the registration information (study registration number, study registration data set designated by WHO, etc.) publicly available.

University Hospital Medical Information Network Research Center Clinical Trial Registration System

（UMIN-CTR、<http://www.umin.ac.jp/ctr/index-j.htm>）

This protocol will be disclosed upon request.

In addition, the results of the study will be made public through conference presentations and paper publication. Post a brief description of the study results for the general public via the hospital web page and SNS, if necessary. Only the investigator(s) who made substantial contributions to the study design, data collection, analysis, or interpretation, was involved in writing or revising the article, and approved the final version of the article, will be identified as the author(s) of the paper. The first author should be the principal investigator. Dissertation writing substitution will not be used.

No public agency or patient will participate in the development of the protocol, execution, or publication of the results of this study.

**１３．Deal with consultations, etc., from research participants and other persons concerned （S31c）**

　Inquiries from research participants will be handled individually by the principal investigator. Inquiries about the research will also be handled by the same contact person.

Organization： Nippon Medical School Musashikosugi Hospital

Address: 1-383 Kosugimachi, Nakahara-ku, Kawasaki-shi, Kanagawa 211-8533

Affiliation: Assistant Professor, Department of Obstetrics and Gynecology

Contact person: Hiroki Shinmura

Phone: 044-733-5181 ext. 3074

E-mail address: h-shimmura@nms.ac.jp

**１４．If the research is to be conducted in a situation where there is an immediate and obvious life-threatening situation for the research subject, how to determine that all of the following four conditions are met**

Research on Antenatal checkup. Not applicable.

**１５．If there is any financial burden or gratuity to the research participants, etc., a statement to that effect and the details of such burden or gratuity（S30）**

No financial burden or honorarium is incurred by the research participants.

**１６．In the case of research involving invasive procedures (excluding minor invasions), what to do in the event of a serious adverse event（S30）**

No medical treatment beyond normal medical practice will be performed in this study. In addition, the study will not include positional guidance that may cause health hazards in the NMS style of postural therapy. There have been no reports of adverse health effects due to the positions taught, either in actual clinical practice or in a literature search. The side-lying position is the first position to be taught to pregnant women during frequent uterine contractions and when discomfort occurs due to the position (e.g., supine hypotension syndrome), and the possibility of health hazards caused by this study, including minor symptoms, is considered to be extremely low. However, any health damage caused by this research will, in principle, be covered by health insurance and treatment will be provided. The treatment provided for any adverse health effects that do occur will be appropriately recorded in the medical record.

**１７． In the case of research involving invasive procedures, whether or not compensation will be provided for any health hazards caused by the research and the details of such compensation（S30）**

In the event that a health hazard occurs to a research participant as a result of this research, the investigators will provide adequate treatment and other appropriate measures, and will make every effort to determine the cause of the hazard. No monetary compensation, such as medical expenses, will be provided. The above will also be stated in the consent form.

**１８．In the case of research involving medical treatment that goes beyond normal medical treatment, actions related to the provision of medical care to research participants after the research is conducted（S30）**

The NMS style of postural therapy does not involve medical treatment beyond the scope of normal medical care. We believe that this is a combination of instructions that is also provided at other facilities within the scope of normal maternal checkups and is not applicable.

**１９．If the conduct of the research may lead to important findings regarding the health of the research participants, genetic characteristics that may be passed on to their pregenerations, etc., the handling of the research results pertaining to the research participants (including incidental findings)**

Not applicable.

**２０．If any part of the work related to the research is outsourced, the nature of such work and the method of supervision of the outsourced party.（S23）**

Not applicable, as all work related to research will be completed within the university.

**２１．If there is a possibility that the sample/information obtained from the research participant will be used for future research that is not specified at the time consent is obtained from the research subject, etc., or will be provided to other research institutions, a statement to that effect and the details expected at the time consent is obtained（S33）**

Not applicable.

**２２．When monitoring and auditing are conducted, the implementation system and procedures（S21a、21b、22、23）**

Although this is an intervention study, we do not believe that a data monitoring committee is necessary because this is only a minor invasion through postural guidance, it is not a long-term study (approximately 3 years), and no serious adverse events are anticipated.

Although a data monitoring committee will not be established, the statistical investigator will conduct an interim analysis only once when the outcome of more than half of the planned number of patients (more than 100) is confirmed, in order to deal with the case in which an effect opposite to the hypothesis, an effect greater than expected, or an unexpected adverse event is observed and the continuation of the study is considered to be detrimental to the research subjects. The significance level of the statistical analysis planned after the completion of the study is p < 0.05, but the interim analysis will be set at p < 0.01. The final decision to discontinue the study will be made in consultation with the study investigators.

The progress of the study and its conformity with ethical guidelines will be checked during the course of the study.

No audits will be conducted, but instead reports will be made to the director of the research institution at least once a year. In addition, if necessary, conformity with ethical guidelines will be checked on each occasion. If a serious adverse event occurs or information is obtained that would compromise the validity of the research, the principal investigator will promptly report this to the director of the research institution, consider measures including early termination of research with co-researchers.

**２３．Approach to Disclosure of Genetic Information**

Not applicable.

**２４．Genetic Counseling System**

Not applicable.

**２５．Name of bank and method of anonymization when providing samples or other materials to a human cell, gene, or tissue bank**

Not applicable.

**２６．Contents of IC when receiving samples or genetic information from other research institutions**

Not applicable.

**２７．Matters such as the method of anonymization when samples or genetic information are provided to an external institution or when a part of the research is entrusted (including the contents of the contract)**

Not applicable.

**２８．Revision History（S3）**

Version 1.0 Created February 28, 2021.

Version 1.1 Created on July 15, 2021. Change of Research Assignee due to retirement, Ethics Committee approval number added, UMIN CTR registration number added.

Version 1.2 Created on September 23, 2021. Change of research institute address due to hospital relocation.

Version 2.0 Created June 08, 2022. Based on the results of the peer review of the protocol paper, the outcome items were revised to more demanding and clinically useful items, and the number of recruits was changed accordingly with a power analysis.

Version 2.1 Created June 09, 2022. With additional details on methods required for practical use.

**２９．References**

1) Chan A-W, Tetzlaff JM, Altman DG, Laupacis A, Gøtzsche PC, Krleža-Jerić K, et al. SPIRIT 2013 Statement: Defining standard protocol items for clinical trials [Japanese]. *Jpn Pharmacol Ther* 2017; 45(12): 1895-1910.

2) ACOG committee opinion, Number 265. Mode of term singleton breech delivery. *Obstet Gynecol* 2006; 108: 235-237.

3) ACOG committee opinion, Number 745. Mode of term singleton breech delivery. *Obstet Gynecol* 2018; 132: e60-e63.

4) Impey LWM, Murphy DJ, Griffiths M, Penna LK on behalf of the Royal College of Obstetricians and Gynaecologists. Management of breech presentation. *BJOG* 2017; 124: e151-e177.

5) Vlemmix F, Rosman1 AN, Fleuren MAH, Rijnders MEB, Beuckens A, Haak MC, Akerboom BMC, Bais JMJ, Kuppens SMI, Papatsonis DN, Opmeer BC, Post JAM, Mol BWJ, and Kok M. Implementation of the external cephalic version in breech delivery. Dutch national implementation study of external cephalic version. *BMC Pregnancy and Childbirth* 2010; 10(20) 1-6.

6) Ducarme G. Breech Presentation: CNGOF Guidelines for clinical practice - external cephalic version and other interventions to turn breech babies to cephalic presentation. *Gynecol Obstet Fertil Senol* 2020; 48(1): 81-94.

7) Hofmeyr GJ, Kulier R. Cephalic version by postural management for breech presentation (Review). *Cochrane Database Syst Rev* 2012; Issue 10. Art. No.: CD000051. DOI: 10.1002/14651858.CD000051.pub2.

8) Coyle ME, Smith CA, Peat B. Cephalic version by moxibustion for breech presentation. *Cochrane Database Syst Rev* 2012; May 16;(5): CD003928. doi: 10.1002/14651858.CD003928.pub3.

9) Schlaeger JM, Stoffel CL, Bussell JL, Cai HY, Takayama M, Yajima H, Takakura N. Moxibustion for cephalic version of breech presentation. *J Midwifery Womens Health* 2018; 63(3): 309-322. doi: 10.1111/jmwh.12752. Epub 2018 May 18.

10) Bue L, Lauszus FF. Moxibustion did not have an effect in a randomised clinical trial for version of breech position. *Dan Med J* 2016; 63(2): A5199.

11) Garcia MM, Gómez DC, Coll MC, Nishishinya B, Allaoui I, Roig G, Tricas GJ. Effectiveness and Safety of Acupuncture and Moxibustion in Pregnant Women with Noncephalic Presentation: An Overview of Systematic Reviews. *Evid Based Complement Alternat Med* 2019; 2019: 7036914. doi: 10.1155/2019/7036914. eCollection 2019.

12) Sananes N, Roth GE, Aissi GA, Meyer N, Bigler A, Bouschbacher JM, Helmlinger C, Viville B, Guilpain M, Gaudineau A, Akladios CY, Nisand I, Langer B, Vayssiere C, Favre R. Acupuncture version of breech presentation: a randomized sham-controlled single-blinded trial. *Eur J Obstet Gynecol Reprod Biol* 2016; 204: 24-30. doi: 10.1016/j.ejogrb.2016.07.492. Epub 2016 Aug 2.

13) Chenia F, Crowther CA. Does advice to assume the kneechest position reduce the incidence of breech presentation at delivery? A randomized clinical trial. *Birth* 1987; 14: 75-78.

14) Hartadottir H, Thornton JG. A randomised trial of the knee/chest position to encourage spontaneous version of breech pregnancies. *Proceedings of 26th British Congress of Obstetrics and Gynaecology* 1992; Manchester, UK, 356.

15) Smith C, Crowther C, Wilkinson C, Pridmore B, Robinson J. Knee-chest postural management for breech at term: a randomized controlled trial. *Birth* 1999; 26: 71-75.

16) Founds SA. Clinical implications from an exploratory study of postural management of breech presentation. Journal of Midwifery & Women's Health 2006; 51(4): 292-296.

17) Bung P, Huch R, Huch A. Is Indian version a successful method of lowering the frequency of breech presentations? *Geburtshilfe und Frauenheilkunde* 1987; 47: 202-205.

18) Obwegeser R, Hohlagschwandtner M, Auerbach L, Schneider B.

Management of breech presentation by Indian version - a prospective, randomized trial [Erhohung der Rate von Spontanwendungen bei Beckenendlagen durch die Indische Brucke? Eine prospective, randomisierte Studie]. *Zeitschri fur Geburtshilfe und Neonatologie* 1999; 203: 161-165.

19) 田岡純次郎. 妊娠中に於ける骨盤位の處置. *産科と婦人科* 1943; 11(10): 40-45.

20） 佐藤純子, 上有谷さつき, 佐藤真紀子, 益鳥杏子, 荒井幸子, 秋元義弘,芳賀健一. 当院における骨盤位の自然修正率と側臥位法による矯正効果の検討. *母性衛生* 1994; 35(3): 207.

21) Brace RA, Wolf EJ. Normal amniotic fluid volume changes throughout pregnancy. *Am J Obstet Gynecol.* 1989; 161(2): 382-388.

22) 吉本英生. 当院における骨盤位の管理 -膝胸位側臥位法および外回転術の有効性についての検討-. *日本周産期・新生児医学会雑誌* 2006; 42(2): 552.

23) Scheer K, Nubar J. Variation of fetal presentation with gestational age. *Am J Obstet Gynecol.* 1976; 125:269.

24) Hickok DE, Gordon DC, Milberg JA, et al. The frequency of breech presentation by gestational age at birth: a large population-based study. *Am J Obstet Gynecol.* 1992; 166:851.

25) Hill LM. Prevalence of breech presentation by gestational age. *Am J Perinatol*. 1990; 7(1): 92-93.

26) Fox AS, Chapman MG. Longitudinal ultrasound assessment of fetal presentation: A review of 1010 consecutive cases. *Aust N Z J Obstet Gynaecol*. 2006; 46(4): 341-344.

図１) ボーアットマーク. “妊婦のイラスト”. 素材ダス. 2021/2/18. http://www.sozaidas.com/forall/fa109hito/fa109hito051.jpg, (2021/2/18)

図２) ボーアットマーク. “妊婦のイラスト”. 素材ダス. 2021/2/18. http://www.sozaidas.com/forall/fa109hito/fa109hito044.jpg, (2021/2/18)

図３) ソーガ運営事務局. “逆子体操をする妊婦さんのイラスト【側臥位（そくがい）法】”. ソーガ. 2021/2/18. https://souga.biz/i1225, (2021/2/18)
