# Supplementary material for: Evaluation of postural therapy using lateral position according to fetal back orientation on breech presentation and breech recurrence (BRLT study): An open-label randomized controlled trial
Source: PLoS Med. 2025 Mar 25;22(3):e1004555. doi: 10.1371/journal.pmed.1004555 (PMC11936219; doi:10.1371/journal.pmed.1004555)
Supplement: S2 Plan — (DOCX) [file pmed.1004555.s005.docx]

**Statistical Analysis Plan**

**１．Title**

**A randomized controlled trial of cephalic version for breech presentation in the third trimester by lateral postural management without knee-chest position (BRLT study)**

Version：1.2

Date：5/14/2022

Protocol Version：2.0

Registration ID：UMIN000043613(3/15/2021)

**２．Author**

Institution (Department)

Nippon Medical School Musashikosugi Hospital Obstetrics and Gynecology

Author’s name, Affiliation, Job title, Role in study

1) principal investigator

Name　 　Hiroki Shinmura

Affiliation, Job title: Obstetrics and Gynecology Assistant Professor

Role: conception, formulation of protocol, data management, data evaluation

2) Statistician

Name　　　　Takashi Matsushima

Affiliation, Job title: Obstetrics and Gynecology Associate Professors

Role: formulation of protocol, data analysis and evaluation

Name　　 　Youhei Tsunoda

Affiliation, Job title: Obstetrics and Gynecology Assistant Professor

Role: formulation of protocol, data collection, data analysis and evaluation

**３．Background**

　The purpose of this study is to verify in a single-center randomized controlled trial whether positional therapy based on the theory of the lateral recumbency method can show an advantage over the control group for the pelvic position in the third trimester. As proof of superiority, the primary endpoint will be the rate of head positioning fetuses at 37 weeks gestation, and whether the percentage of fetuses converted from pelvic to head position is significantly higher in the instructed group compared to the control group at 37 weeks gestation will be tested. At the same time, the cesarean section rate, head position rate after 2, 4 and 6 weeks of instruction, breech recurrence rate, and adverse events will also be evaluated as secondary endpoints.

　This statistical analysis plan will detail the analytical methods to evaluate these endpoints. If there are any changes in data handling or analysis methods, this plan may be revised each time upon consultation among the investigators.

**４．Design of study**

　This is an open-label, parallel, randomized, controlled trial of the superiority of the NMS-style postural therapy. The allocation ratio is intervention group　vs. control group = 1:1.

**５．Study Participants**

The inclusion criteria are as follows:

・Breech presentation diagnosed on screening ultrasound examination

・Gestational age between 28+0 and 30+0 weeks

・Pregnant women undergoing prenatal check-ups at the Nippon Medical School Musashikosugi hospital

・Over 20 years old

Exclusion criteria were as follows:

・already receiving treatment for pelvic position

・placental malposition

・previous cesarean section,

・scheduled cesarean section after uterine myoma surgery, etc.

・scheduled delivery at another hospital

・multiple pregnancies

・transverse position

・complications considered risky for performing positional therapy (such as history of heart disease)

　If determined to be eligible, they would be invited to participate in the study and consent would be obtained at that day.

**６．Enrollment and Randomization**

　If determined eligible, the patient will be invited to participate in the study at that time and written consent will be obtained. Once written consent is obtained, the participants will be randomly assigned to the intervention and control groups using an allocation table provided in the outpatient clinic. The allocation table will be created by an independent researcher using a random function and random block sizes of 4, 6, and 8. There will be two allocation tables, one for first-time mothers and the other for postpartum women, to allow for stratification between the two groups (parity has a significant impact on pelvic presentation treatment, Levin et al. 2019). The allocation tables are sealed and confidential, and the order cannot be predicted until the seal is removed. Assignments are made by an outpatient staff member, independent of the researcher, who removes the stickers in order from the allocation list. The allocation group is disclosed to the participant and the researcher, but the allocation group is kept secret from the statistician during statistical analysis.

**７．Intervention and Measurement**

　The intervention group will be instructed in the Nippon Medical School style of positional therapy. The lateral position is taught for the pelvic position and the reverse lateral position for the head-first position, and the direction of lying is determined by the position of the fetal back. In the intervention group, participants are instructed to perform postural therapy three times daily for 15 minutes each. In the control group, the usual maternal care will be provided according to the Japanese obstetric guidelines. In both groups, ultrasound screening is performed at the time of enrollment, followed by ultrasound examinations every 2 weeks to record fetal presentation, orientation, fetal back position, estimated fetal weight, and amniotic fluid pocket. The lying orientation is recorded on a recording form by all and collected every 2 weeks. At the time of enrollment, age, gestational age, height, weight, placental position, infertility treatment, pre-existing complications and obstetric complications are obtained from the electronic medical record. Adverse reactions to the positional therapy are reported on a case-by-case basis. At the time of delivery, delivery weeks, fetal presentation, method of delivery, birth weight, Apgar score, umbilical artery blood pH, and volume of blood loss at delivery are obtained from the electronic medical record.

**８．Objective**

　The Aim of this trial is to evaluate whether the instruction of lateral position can reduce fetuses in breech presentation in women between 28+0 and 30+0 weeks of gestation, compared with expectant management care.

**９．Outcome**

**① Primary outcome**

　The reduction of breech fetuses will be determined using the rate of cephalic fetuses in each group at 37 weeks gestation.

**② Secondary outcomes**

　The secondary outcomes are cephalic presentation two, four, and six weeks later, cesarean delivery, caesarean delivery for breech presentation, recurrent breech presentation after cephalic version, and adverse effects. Only the recurrent breech position will be compared between groups converted to a cephalic presentation at least once.

**１０．Sample Size Calculation**

　The sample size was calculated based on our previous retrospective cohort study. In our previous study, the head-first position rate was 94% (16/17) in the lateral position group and 77% (30/39) in the control group (Shinmura et al. 2021). Therefore, in this study, the expected percentage of head-first position in the lateral position intervention group was 94%. Normally, 20-25% of fetuses are in the pelvic position at less than 28 weeks, dropping to 3-4% in the term group, and the head-first position conversion rate is around 84% (Scheer et al. 1976, Hickok et al. 1992). Furthermore, in a study of 1,010 patients, Fox et al. reported a 75% probability that a fetus who was in the breech position at 28 to 30 weeks would return to the cephalic position at term (Fox et al. 2006). Based on the above evidence, the predicted head-first position conversion rate at term in the control group was 80%. In this study, the effect size was set at 94% for the intervention group and 80% for the control group, using G*Power (version 3.1, Faul, Erdfelder, Lang, and Buchner, Düsseldorf, Germany) with error = 0.05 and 1-β error = 0.80, The effect size φ = 0.2081 was calculated. Considering the missing value to be approximately 10%, we selected 100 participants per arm, making a total of 200 participants. Secondary endpoints were not considered in the sample size calculation.

**１１．Trial Duration**

　In our previous retrospective cohort study, 70 participants were included in the study in 12 months. This trial is expected to take approximately three years to reach the planned sample size of 200. Therefore, the planned study period was set from April 1, 2021 to March 31, 2024.

**１２．Analysis**

　　The analysis will be conducted after the scheduled participants have been included, data collection for each outcome has been completed, and the data have been fixed. Summary statistics are calculated for each group for each outcome. In principle, the analysis is performed without excluding any abnormal data. The primary and secondary outcomes are binary categorical variables, and comparisons between the two groups are made with the χ2 test (Pearson's test or Fisher's test). The unadjusted risk ratio is calculated as the main outcome along with a 95% confidence interval. Absolute and relative risk reductions are calculated simultaneously. If the continuous variables are normally distributed, a Student's t-test is performed; if not normally distributed, a Mann-Whitney U test is performed; if there is bias in the characteristics between the two groups, the analysis is also adjusted for that factor as a confounding factor.

**① Intention-to-treat analysis**

　　The intention-to-treat analysis, which is treated as the primary outcome, includes protocol deviations and compares all participants with results between the two groups.

**② Per-protocol set analysis (PPS)**

　　Already in the planning stages of this study, the first randomized controlled trial to examine lateral position management for breech presentation, which has never been tested at a high level of evidence, we felt that the presence of a potential lateral position group in the control group would be an obstacle to answering the pure question, “Can head conversion really be achieved simply by lying on the side?” Therefore, we considered it necessary to conduct a per-protocol set (PPS) analysis excluding the potential intervention group within the control group in order to test the pure efficacy of the lateral position, apart from the clinical efficacy of the intention-to-treat analysis. A patient will be considered to have achieved lateral position success if she is able to lie on her side in the correct orientation for at least 45 minutes during at least half of the 2-week period. Whether patients were able to perform the postural therapy according to the protocol will be recorded in a categorical variable; the PPS analysis will compare the intervention group, in which cases with a lateral position success rate of less than 50% will be excluded, with a control group, in which cases with a lateral position correct rate of more than 50% and similar posture as the intervention group will be excluded. Note that due to the nature of the lateral position method, it was impossible to prohibit the lateral position in the control group participants, and although it was conceivable that spontaneous and intentional crossovers could occur, the analysis will be conducted according to the protocol predetermined in this plan. In the event of a large number of deviants, we will also evaluate for attrition bias.

**③ Subgroup Analysis**

　Subgroup analyses will be conducted separately for first-time mothers and multiparous mothers. Because of the small sample size of the subgroup analysis, which is expected to reduce the power of the analysis from the outset, tables and other information will be provided in an Appendix rather than in the main text of the paper.

**④ Stratified analysis**

　Since we are stratifying for parity, we perform a Cochran and Mantel-Henzel test as a stratified analysis.

**⑤ Adverse Events**

　Adverse events will be reported by participants on a case-by-case basis, and their incidence will be compared with that of the control group. In principle, the same groups will be evaluated as in the intention-to-treat analysis.

**⑥ Missing Values**

　For missing values, we will use mean imputation for continuous variables and pairwise deletion for categorical variables. Whenever they are used, they will be reported in the paper.

　Currently, there are a few missing values for amniotic fluid volume. Mean replacement is used for these. If the variables are normally distributed, the mean value is substituted; if the variables are non-normally distributed, the median value is substituted.

**⑦ Significance level**

　A p-value <0.05 is considered statistically significant, and a two-sided test is used in all analyses.

**⑧ Statistical Software**

Statistical Package for the Social Sciences software for Windows (version 26.0, IBM Corp.) will be used for statistical analysis.

**⑨ Interim Analysis**

The statistical investigator will conduct an interim analysis only once when the outcome of more than half of the planned number of patients (more than 90) is confirmed, in order to deal with the case in which an effect opposite to the hypothesis, an effect greater than expected, or an unexpected adverse event is observed and the continuation of the study is considered to be detrimental to the research subjects. The significance level of the statistical analysis planned after the completion of the study is p < 0.05, but the interim analysis will be set at p < 0.01. The final decision to discontinue the study will be made in consultation with the study investigators.

**１３．Revision History**

Version 1.0 Created March 26, 2021 based on Protocol Version 1.0.

Version 1.1 Revised September 21, 2021 with additional details after the study began.

Version 1.2 Revised May 14, 2022 based on Protocol Version 2.0

　　　　　　Changes made to outcome and sample size calculations
