# Supplementary material for: Evaluation of postural therapy using lateral position according to fetal back orientation on breech presentation and breech recurrence (BRLT study): An open-label randomized controlled trial
Source: PLoS Med. 2025 Mar 25;22(3):e1004555. doi: 10.1371/journal.pmed.1004555 (PMC11936219; doi:10.1371/journal.pmed.1004555)
Supplement: Attachment — Submitted filename: Reply_to_Comments.docx [file pmed.1004555.s009.docx]

**Our responses to the editors’ and the reviewers’ comments**

We would like to thank the editors for inviting us to revise our manuscript. We also appreciate the reviewers for the valuable time and effort you put into our manuscript. We have revised the manuscript in accordance with your generous and helpful comments. We also hope that our revisions and the responses below will satisfactorily address all the issues and concerns you have noted.

**Editors:**

We are grateful for the opportunity to revise our manuscript.

**> We note some discrepancies between the original protocol and this report of the findings from the trial.**

As you indicated, we changed the primary endpoints and the number of recruits in the course of this study. This is because during the peer review of the protocol paper, a reviewer strongly recommended that the original primary endpoint of cephalic version rate after 2 weeks be clinically unimportant and that the clinically important cephalic version rate at term (which was initially a secondary endpoint) be the primary endpoint. We discussed this and carefully considered. A power analysis of the case with the primary endpoint of cephalic version rate at term showed that the increase in recruitment was well within the feasible range, so the decision was made to change the primary endpoint and the number of recruits, and the Ethics Committee approved and we changed the content of the trial registry.

**> 1) Primary and secondary outcomes**

**> We note that the primary endpoint in protocol was specified as the rate of head conversion 2 weeks after teaching postural therapy, whereas the primary endpoint in manuscript was % of breech presentation cases at 37 weeks gestation. It appears that primary and secondary outcomes with relation to % breech at 37 gestational weeks and head presentation at 2 weeks after the intervention have been switched.**

**> Please provide details of any protocol amendments to justify changes to the primary outcomes of the trial. Please provide documentary evidence that the changes were approved by a steering committee / IRB.**

As explained above, the primary endpoint was changed during the course of the study. This is noted in the revision history of the latest protocol version 2.1, which we will be submitting. We will also provide documentation that this amendment has been approved by the IRB and the hospital director.

**> 2) Power calculation**

**> We note that the predicted conversion rates changed in the power calculation. In the protocol, it was assumed that there would be 80% and 60% conversion in intervention and control arms respectively. In the manuscript, it is assumed that there would be 94% and 80% conversion in intervention and control arms respectively. Originally, n=165 were required per group. In the manuscript, n=100 are required per arm.**

**> Please provide justification for the changes made to the above assumptions and sample size. If amendments to the trial protocol and SAP were made after the original versions of these documents were finalised, please provide copies of the final versions of the protocol and SAP as a supplementary file alongside your revised manuscript. If the SAP differs from the protocol, please justify any changes.**

The power was reanalyzed to reflect the changes in the primary outcome described above. The number of participants was changed according to the analysis results, and the trial registration details were changed after the protocol amendment was approved. The statistical analysis plan was updated accordingly. We will also submit the revised statistical analysis plan along with the manuscript.

**> In addition, in line with comments from the reviewers and the Academic Editor, please provide a copy of the original (raw) data in your revised version.**

Raw data will also be submitted as requested by the academic editor and the third reviewer.

**Reviewer #1:**

We sincerely thank you for your review and helpful comments on the statistical analysis. We have made further corrections and considered some additional analyses in accordance with your comments. Details are provided below.

**> First, the primary outcome data are presented as the percentage of women with a breech presentation at 37 weeks being reduced in the intervention arm: 11% vs 19%. Since the risk in the intervention arm is lower, this appears as a RR<1, namely 0.91. The problem is that 11/19 = 0.58. It appears that the authors have calculated the RR as the risk of not having the primary outcome in the control arm (81%) divided by the risk of not having the primary outcome in the intervention arm (89%). This seems to happen for many of the outcomes, but is not entirely consistent. In general, when talking of relative "risk", that implies looking at the probability of the adverse outcome. Also, when comparing treatment groups, one normally reports the risk in the intervention arm divided by the risk in the control arm, so that if the risk is reduced, the RR is less than 1.**

Thank you for your important remarks. We have reviewed all relative risks and have revised the main text and all tables, dividing the risk in the intervention group by the risk in the control group, as you have indicated.

On page 3, lines 43–45, 48–52.

Breech presentation at 37 gestational weeks was reduced by approximately 1/2 through the combination of the opposite-side and same-side lateral positions in the intervention group compared with that in the control group. However, we found no statistical significance in the intention-to-treat analysis (11% [11/100] vs. 19% [19/100]; absolute risk difference, −8.0% [95% CI, −17.8%–1.84%]; relative risk, 0.58 [95% CI, 0.29 to 1.15]; p = 0.11). The per-protocol analysis found the same direction of effect but with statistical significance. In the intention-to-treat analysis, the intervention group had a higher cephalic version rate 2 weeks after the instruction (69% [69/100] vs. 54% [54/100]; absolute risk difference, −15.0% [95% CI, −28.3% to −1.67%]; relative risk, 0.67 [95% CI, 0.47 to 0.96]; p = 0.029), and lower breech presentation recurrence rates (2% [2/91] vs. 10% [9/88]; absolute risk difference, −8.0% [95% CI, −15.0% to −1.02%]; relative risk, 0.22 [95% CI, 0.048 to 0.97]; p = 0.031) than the control group. In the intervention group, three participants experienced discomfort and one participant complained of pain in the lateral abdomen, but these symptoms resolved spontaneously.

On page 24, lines 386–387.

As the primary outcome in the intention-to-treat analysis, breech presentation occurred at 37 weeks in 11 participants (11% [11/100]) from the intervention group and 19 (19% [19/100]) from the control group; thus, breech presentation occurred in almost a half of those in the intervention group. However, the difference was not statistically significant (absolute risk difference, −8.0% [95% CI, −17.8% to 1.84%]; relative risk, 0.58 [95% CI, 0.29 to 1.15]; p = 0.11; Table 2).

On page 24, lines 392–394.

Two weeks after the start of the positioning instruction, breech presentation was converted into cephalic in 69% (69/100) of the participants in the intervention group; this percentage was significantly more than that in the control group (54% [54/100]) (absolute risk difference, −15.0% [95% CI, −28.3% to −1.67%]); relative risk, 0.67 [95% confidence interval 0.47 to 0.99]; p = 0.029).

On page 24, lines 396–398.

After 4 or 6 weeks of instruction, the cephalic version rate did not significantly differ between such groups. After cephalic version, the intervention significantly prevented breech presentation recurrence more than the control (2% [2/91] vs. 10% [9/88]; absolute risk difference, −8.0% [95% CI, −15.0% to −1.02%]; relative risk, 0.22 [95% CI, 0.048 to 0.97]; p = 0.031).

On page 25, lines 404–405.

Moreover, the overall cesarean section rate was significantly lower in the intervention group than in the control group (23% [23/100] vs. 36% [36/100]; absolute risk difference, −13.0% (95% CI, −25.5% to −0.49%); relative risk, 0.64 (95% CI, 0.41 to 0.99); p = 0.044), although this result included one patient with a previous cesarean section.

On page 25, lines 414–415.

Using a predetermined method, the per-protocol analysis revealed that the intervention group had a statistically significant reduction in pelvic position at 37 weeks (9% [8/90] vs. 21% [15/72]); absolute risk difference, −11.9% [95% CI, −23.0% to −0.87%]; relative risk, 0.43 [95% CI, 0.19 to 0.95]; p = 0.030; Table 3).

On page 26, lines 426–432.

In the subgroup analyses restricted to multiparous women, the intervention group had a significantly higher rate of cephalic version after 2 weeks (86% [31/36] vs. 50% [19/38]); absolute risk difference, −15.6% [95% CI, −29.1% to −2.20%]; relative risk, 0.28 [95% CI, 0.02 to 0.67]; p = 0.001) and after 6 weeks (97% [35/36] vs. 79% [30/38]; absolute risk difference, −18.3% [95% CI, −32.3% to −4.25%]; relative risk, 0.13 [95% CI, 0.017 to 1.00]; p = 0.029), and a lower rate of caesarean section (6% [2/36] vs. 26% [10/38]); absolute risk difference, −20.8% [95% CI, −36.6% to −4.89%]; relative risk, 0.21 [95% CI, 0.05 to 0.90]; p = 0.015) than the control group (S1 Table).

On pages 25–26, Table 2, 3.

Tables 2, 3, S1, and S2.

Table S1, Table S2.

**> Secondly, the authors present per-protocol analyses. This is commonly done, but is not without problems, since the population for analysis is based on participants' behaviour after being randomised, giving a biased estimate of the effect of the intervention. Nowadays, there are better methods, such as Complier Average Causal Effects (CACE) methods, which can estimate the effect of the intervention, allowing for non-compliance in the intervention arm, as well as a proportion of the control arm receiving the intervention. If possible, it would be good to provide CACE estimates of intervention effects. Since this was not pre-specified, it would be fine to put this into a supplement.**

Thank you for your excellent suggestions. We estimated the effect of the intervention by using the Complier Average Causal Effects (CACE) method, considering the noncompliance in the intervention group and the proportion of the control group that could be considered as having received the intervention. The calculated CACE estimates are mentioned in the Supplementary Appendix.

Supplementary Appendix, Complier Average Causal Effects (CACE) Method.docx

**> Another thing that could be a little better is the subgroup analysis of primiparous vs multiparous women. Currently, the analysis is done separately in the two subgroups, and a significant result is shown for multiparous women, but not for the primiparous group. This is interesting, but on its own, does not allow any conclusions to be drawn. It is better to do an analysis that formally tests whether the intervention effects in the two subgroups are the same or different. For example, a logistic regression model with an interaction between parity and the intervention would achieve this. Only if this interaction test shows some evidence of a difference does it become reasonable to interpret the separate intervention effect estimates. Since the study was designed to detect an overall intervention effect, any interaction test will be underpowered, but this would be a more robust approach.**

In line with your suggestion, we have conducted the logistic regression analysis with interactions between parity and intervention. This analysis is described in the subgroup analysis section.

On page 27, lines 434–443.

We conducted logistic regression analysis to test whether the intervention effects were the same or different between two subgroups, namely, primiparous and multiparous women. We used breech presentation at 37 gestational weeks as the dependent variable. Covariates included the presence or absence of parity and the presence or absence of intervention. In the set of intention-to-treat analyses, the odds ratio of having intervention was 0.51 (95% CI, 0.23–1.15; p = 0.11) for the control group; in the set of per-protocol analyses, the odds ratio of having intervention was 0.37 (95% CI 0.15–0.94, p=0.036) for the control group. The trend of the effect was similar to the results of the univariate analysis.

**> Line 290-291 states that one women was excluded from the PP dataset due to a planned caesarean at 38 weeks. Why, if the primary outcome was measured at 37 weeks?**

One patient had a scheduled cesarean section, which is an exclusion criterion. Whether or not the breech presentation is corrected in such an individual is considered clinically meaningless and a serious deviation from the protocol. Therefore, they were excluded from the per-protocol analysis.

**> The section on subgroups analyses only reports p-values. As stated above, an interaction test of some sort should be included, but when presenting the results within subgroups, the intervention effect estimates and confidence intervals should be reported. Also, would it be possible to show the subgroup analyses in a forest plot? Maybe it wouldn't work, because this is usually done when there is one (primary) outcome and several subgroup analyses; here we have multiple outcomes, looked at in the same subgroups.**

As indicated, we have revised the subgroup analysis section to show the intervention effect estimates and 95% CI along with the p-values. In accordance with your suggestion, we have attempted to create a forest plot, but we eventually decided against it because we thought it would be too many figures, and conversely, it would be difficult to understand. We apologize for not following your intention earlier.

On page 26, lines 426–432.

In the subgroup analyses restricted to multiparous women, the intervention group had a significantly higher rate of cephalic version after 2 weeks (86% [31/36] vs. 50% [19/38]); absolute risk difference, −15.6% [95% CI, −29.1% to −2.20%]; relative risk, 0.28 [95% CI, 0.02 to 0.67]; p = 0.001) and after 6 weeks (97% [35/36] vs. 79% [30/38]; absolute risk difference, −18.3% [95% CI, −32.3% to −4.25%]; relative risk, 0.13 [95% CI, 0.017 to 1.00]; p = 0.029), and a lower rate of caesarean section (6% [2/36] vs. 26% [10/38]); absolute risk difference, −20.8% [95% CI, −36.6% to −4.89%]; relative risk, 0.21 [95% CI, 0.05 to 0.90]; p = 0.015) than the control group (S1 Table).

**> The methods section says that results are reported as relative risks, and absolute risk differences (as they should, according to CONSORT), but I do not recall seeing the absolute risk differences presented or discussed anywhere.**

As you have highlighted, none of the results showed an absolute risk difference, except for the primary endpoint of -8%. For the other results, we have indicated the absolute risk difference and the relative risk with 95% CIs. We considered adding absolute risk differences to the table but eventually decided against it because it seemed to make the table too busy and confusing.

On page 3, lines 43–45, 48–52.

On page 24, lines 386–387, 392–393, 396–398.

On page 25, lines 404–405, 414–415.

On page 26-27, lines 426–432.

**Reviewer #2:**

I am grateful for your careful review of our paper despite its offensive nature, and for your helpful suggestions. Based on your suggestions and comments, after careful consideration, we have made significant revisions regarding background and conclusions. We have also decided to adopt your suggestion and make significant changes to the terminology we use. Details are described below.

**> Overall, my main discomfort with the paper is the authors' continued use of the terms 'validated.' The study did not demonstrate a statistically significant difference in the primary outcome. It has provided data that suggest further research is warranted. But much more research is warranted before this can be recommended. In many settings, it would require introducing interventions (eg. scans, advice) that are not currently used at earlier gestations.**

As you have highlighted, this study did not find a significant difference in the primary endpoint in the intention-to-treat analysis. Therefore, the word “validated” is inappropriate and has been revised accordingly. Furthermore, we agree that further research is needed before recommending this treatment. Nonetheless, this study provides a new concept of breech presentation treatment, although certain factors need to be considered in its implementation. One of these factors is the timing of ultrasound examinations, and it varies from country to country.

On page 2, line 28.

Few studies have tested lateral position management for breech presentation, and no method exists to prevent breech recurrence after cephalic version.

On page 18, line 318.

Already in the design phase of this study, the first randomized controlled trial to examine the lateral position management which has never been tested with a high level of evidence.

On page 29, line 470.

Currently, no randomized controlled trials have tested the lateral position management for breech presentation, and all trials that have validated postural therapy for breech presentation have been related to the knee–chest position or the deep Trendelenburg position [11-14].

On page 31, line 503.

In the same side lateral position (modification of the opposite side lateral position) performed after cephalic version, gravity is used to stabilize the fetus; however, its effectiveness has never been tested.

**> This RCT has been performed on a sample of women having routine ultrasound scanning at 28 to 30 weeks. Its applicability outside of Japan is therefore contained to settings which perform routine USS at 28 to 30 weeks, and who begin a breech care pathway at this point if identified. This would not include the UK, where routine ultrasound scanning is not performed at this time, and where the usual NHS breech care pathway does not begin until 36 weeks. There is very little likelihood that an intervention requiring an USS every two weeks from 30 weeks would be implemented.**

The intervention for this study was set up to begin at 28–30 gestational weeks, which is usually the time of second ultrasound screening in the third trimester of pregnancy in Japan. We acknowledge that the timing and frequency of ultrasound scans vary from country to country, and the dilemma is that starting intervention earlier than this period may result in overtriage and performing intervention immediately before full-term labor may not be effective enough. Thus, the timing of the start of the intervention remains debatable.

**> Caesarean birth is also performed at 39 weeks in the UK and most countries, due to the preference for fetal lung maturity. Again, this brings into question the applicability to international practice.**

In many facilities in Japan, scheduled cesarean sections are still performed at 38 gestational weeks, and we have followed this practice in this study. However, waiting until 39 weeks for cesarean section may be reasonable and may reduce unnecessary cesarean sections, given that spontaneous fetal rotation can be expected.

**> Having read the article, I find the terms 'lateral position' and 'reverse lateral position' difficult to understand and counter-intuitive. For example, in reverse lateral position, the mother is lying on the SAME side where the baby's back lies. In lateral position, it is the OPPOSITE side as the baby's back. These terms seem the opposite of what I would assume.**

**>To English language speakers, the terms 'same side lateral position,' meaning same side as the baby's back, and 'opposite side lateral position,' meaning opposite side as the baby's back, may make more sense. I would encourage the authors to reach out to an international audience and sense-check these terms.**

Thank you for your excellent suggestions. We also, had concerns about the terminology setup. In Japan, the term “lateral position” generally refers only to the “opposite side lateral position” as you have highlighted, and our reverse lateral position is not known. We were setting up the terminology according to Japanese convention, but we find your suggestion of “opposite side lateral position” and “same side lateral position” to be considerably intuitive and comprehensible. Native speakers also commented that your proposal was easy to understand. After discussion with the co-authors, we have decided to change “lateral position” to “opposite side lateral position” and “reverse lateral position” to “same side lateral position” in this paper. We believe that these terms are easy to understand.

**> "Few studies have validated lateral position management for breech presentation …" This assumes that readers know what lateral position management means. We don't. Try using plain English to describe what you are doing, from the start.**

Thanks for pointing this out. For clarity, we have added an explanation of the lateral position method.

On page 2, lines 26–27.

In Japan, the lateral position method is known as a postural therapy for breech presentation wherein the mother lies down in lateral position according to fetal back. Few studies have tested lateral position management for breech presentation, and no method exists to prevent breech recurrence after cephalic version.

**> The concluding statements are not warranted. The study did not demonstrate a statistically significant difference in the primary outcome, breech presentation at 37 weeks. The direction of effect is promising, but this can only be considered a pilot study, not a basis for clinical recommendations. Also, the study did not aim to assess the effect of 'reverse lateral position' as a maintenance therapy. Again, recommendations are not warranted.**

As you indicated, the result did not lead to treatment recommendation, although it indicated the possibility of this study treatment. The conclusions have been substantially revised.

On pages 4, lines 56–65.

For breech presentation at the beginning of third trimester, providing a combined instruction regarding opposite side lateral position for breech presentation and same side lateral position after cephalic version reduced the number of breech fetuses at 37 gestational weeks by approximately one-half; however, this was not showing statistical significance. In the secondary endpoints, the intervention group showed significantly high rates of cephalic version after 2 weeks and low rates of breech recurrence. The direction of the effect was promising, and this study provides a new concept for breech presentation treatment, that is, reducing breech presentation and preventing breech recurrence simply by lying down according to fetal back. To validate this concept, further research with consideration for unplanned crossover is warranted.

On page 34-35, lines 556–563.

Regarding the secondary outcomes, the lateral positional group had a considerably high rate of cephalic version after 2 weeks of instruction and a low rate of breech recurrence. The direction of these effects is also promising, and this trial presents a new treatment concept for breech presentation, that is, correcting breech presentation and preventing breech recurrence simply by lying down according to fetal back. Although the application of the lateral position method before and after external cephalic version is assumed, further research considering the impact of unscheduled crossover is warranted.

**> p.4, line 58. 'Vaginal delivery in breech presentation is associated with perinatal morbidity and mortality.' - This statement needs a lot more specificity. Pregnancy, and any type of birth, is associated with perinatal morbidity and mortality.**

Thank you for pointing this out. We have corrected the unclear statement.

On page 6, lines 96–98.

Breech presentation occurs in 3%–4% of all term pregnancies, and vaginal delivery in breech presentation is associated with perinatal morbidity and mortality compared with planned cesarean section.

**> Line 59 - Caesarean section was recommended by whom? ACOG? This isn't the most recent guidance. Is it the guidance followed in Japan?**

Thank you for highlighting this. The wording was indeed misleading. We have now corrected the description for clarity.

On page 6, line 98–100.

Since a large randomized controlled trial published by Hannah et al. (2000), cases of cesarean section for breech presentation have increased worldwide.

**> Line 61 - 'The only established treatment for breech presentation is external cephalic version.' What does this mean? External cephalic version is not the only way to 'treat' breech presentation. There are many methods of delivering breech babies vaginally as well.**

We agree that stating that it was the only treatment is incorrect. Therefore, we have correct this in the revised manuscript.

On page 7, lines 105.

External cephalic version is an established treatment method for breech presentation, which is performed after 36 gestational weeks, but it poses problems such as pain and unsuccessful cases.

**> Listing potential 'treatments' for breech presentation before 36 weeks and saying their effectiveness 'remains controversial,' is vague. This paper should include a scientific summary of the availably literature.**

We agree that the explanation was unclear. The sentence has been corrected to be more specific.

On page 7, line 110.

Conversely, treatment for breech presentation before 36 weeks has not yet been established. Postural therapies, such as knee–chest position and deep Trendelenburg position, or acupuncture and moxibustion are known treatments that can be performed before 36 weeks; however, evidence to recommend these treatment remains insufficient.

**> Line 68 - "In 1812, Wigand described the lateral position as effective when the fetal head descended from the transverse position with the fetal back on the fundal uterine side." - I do not understand what this means at all. What does 'lateral position' mean - which lateral position? What does 'fundal uterine side' mean? This paper requires clearer explanations.**

We have respected the original statement by Wigand; however, we apologize for any difficulty in understanding it. We have re-explained it in a straightforward manner for clarity and understanding.

On page 7, lines 112–113.

Wigand (1812) described the lateral position as effective when correcting oblique position to cephalic position.

**> I have scrolled to the bottom and identified the figures with the lateral positions. These need to be used very early on in the paper, so that readers have a common understanding of terms used. 'Lateral position' and 'reverse lateral position' should be defined, with figures, from the very beginning.**

Thank you for highlighting this. Indeed, it was difficult to understand. Therefore, we have modified the background so that the figure can be referenced as early as possible.

On page 7, lines 113–116.

The lateral position on the opposite side of fetal back for breech presentation (Fig 1, opposite side lateral position, A and B), which is a modification of Wigand’s method, has been performed along with the knee–chest position in Japan since 1943, when this report was published by Taoka (1943).

On page 8, lines 123–124.

Its effectiveness had never been verified, but the lateral position on the same side of fetal back (Fig 1, same side lateral position, C and D) was theoretically a safe method for preventing breech presentation recurrence after cephalic version.

**> I would like to openly declare my biases: I research techniques to make vaginal breech birth safer. I have been working in breech clinics and researching breech care for 14 years. I am aware of how much time and effort it takes to 1) identify breech presentations; 2) counsel women about even position changes; and 3) monitor outcomes. There are economic costs to all of this, both to services and to women and families, due to needing to present for healthcare (societal costs).**

**> Therefore, before any recommendations can be made, definitive trials need to be conducted, and these need to have an economic component.**

**> I would encourage the authors to consider becoming aware of recent developments in vaginal breech delivery as well, and whether this impacts whether such early, frequent assessment and intervention in breech presentation is cost- and clinically effective.**

We would like to express our respect for your commitment to care for vaginal deliveries in the breech presentation. We know that it requires a lot of work. We are also very interested in the costs and economic factors involved in therapeutic interventions. In this study, fetal positioning was confirmed using a stationary ultrasound device during follow-up every 2 weeks to ensure the highest priority on accuracy. However, using the stationary ultrasound equipment every time is costly. Thus, considering the possibility of replacing stationary ultrasound systems with the Leopold palpation method and small portable ultrasound systems is necessary. We also need to evaluate how well the positive diagnosis rate of the Leopold palpation method is comparable with that of ultrasonography and whether it can be used for practical purposes. Recently, the performance of small portable ultrasound systems has improved, and their prices have come down, making them a promising alternative. We will continue to explore ways to optimize the method of fetal location, including the gestational age (in weeks) of confirmation, confirmation intervals, and the means of confirmation.

On page 6, lines 100–116.

Conversely, a cohort study showed that under the guidance of experienced physicians, vaginal delivery in breech presentation is as safe as cesarean sections, and there is a movement to rethink vaginal delivery in breech presentation [6].

**Reviewer #3:**

Thank you for your generous feedback. We have revised the paper according to your suggestion.

**> One suggestion is to have a native speaker with knowledge of clinical trials to edit the manuscript.**

We had an experienced native English speaker with knowledge of clinical trials proofread our revised paper.

**> I miss table with obstetric outcomes: gestational age at delivery, delivery mode, birth weight, Apgar scores at 1 and 5 minutes, umbilical artery pH at birth, bleeding amount during delivery, and perinatal complications.**

**This should all be tabulated.**

We have prepared a table of obstetric outcomes in accordance with your instruction.

On page 28, lines 453, Table 4.

**> I would like to see the raw data underlying the paper.**

We would be submitting the raw data as per your request.

**> Minor issue: Inclusion criteria: " gestational age between 28+0 and 30+0 gestational weeks". Results: The median number of gestational weeks at randomization was 28 weeks for all groups. Table says 28+6.**

The reason for this is unclear to us. We do not have any special appointment management; however, we suspect that the nature of our hospital’s maternity management schedule has an impact. In principle, all women who met the eligibility criteria at the number of eligible gestational weeks were invited to participate in the study.

**General editorial requests:**

We have confirmed and responded to the requirements stated.

**> *	We ask every co-author listed on the manuscript to fill in a contributing author statement, making sure to declare all competing interests. If any of the co-authors have not filled in the statement, we will remind them to do so when the paper is revised. If all statements are not completed in a timely fashion this could hold up the re-review process. If new competing interests are declared later in the revision process, this may also hold up the submission. Should there be a problem getting one of your co-authors to fill in a statement we will be in contact. Please do not add or remove authors without first discussing this with the handling editor. You can see our competing interests policy here: http://journals.plos.org/plosmedicine/s/competing-interests.**

All authors declared that they have no competing conflicts of interest and declared this in the submission system.

**> *	Please upload any figures associated with your paper as individual TIF or EPS files with 300dpi resolution at resubmission; please read our figure guidelines for more information on our requirements: http://journals.plos.org/plosmedicine/s/figures. While revising your submission, please upload your figure files to the PACE digital diagnostic tool, https://pacev2.apexcovantage.com/. PACE helps ensure that figures meet PLOS requirements. To use PACE, you must first register as a user. Then, login and navigate to the UPLOAD tab, where you will find detailed instructions on how to use the tool. If you encounter any issues or have any questions when using PACE, please email us at PLOSMedicine@plos.org.**

We have confirmed the requirements stated.

**> *	Please ensure that the paper adheres to the PLOS Data Availability Policy (see http://journals.plos.org/plosmedicine/s/data-availability), which requires that all data underlying the study's findings be provided in a repository or as Supporting Information. For data residing with a third party, authors are required to provide instructions with contact information (web or email address) for obtaining the data. Please note that a study author cannot be the contact person for the data. PLOS journals do not allow statements supported by "data not shown" or "unpublished results." For such statements, authors must provide supporting data or cite public sources that include it.**

We have confirmed the abovementioned requirements.

**> *	We expect all researchers with submissions to PLOS in which author-generated code underpins the findings in the manuscript to make all author-generated code available without restrictions upon publication of the work. In cases where code is central to the manuscript, we may require the code to be made available as a condition of publication. Authors are responsible for ensuring that the code is reusable and well documented. Please make any custom code available, either as part of your data deposition or as a supplementary file. Please add a sentence to your data availability statement regarding any code used in the study, e.g. "The code used in the analysis is available from Github [URL] and archived in Zenodo [DOI link]" Please review our guidelines at https://journals.plos.org/plosmedicine/s/materials-software-and-code-sharing and ensure that your code is shared in a way that follows best practice and facilitates reproducibility and reuse. Because Github depositions can**

**be readily changed or deleted, we encourage you to make a permanent DOI'd copy (e.g. in Zenodo) and provide the URL.**

We have confirmed the requirements stated.

FORMATTING – GENERAL

**> *	Abstract: Please structure your abstract using the PLOS Medicine headings (Background, Methods and Findings, Conclusions). Please combine the Methods and Findings sections into one section.**

We have confirmed the requirements stated above.

**> *	At this stage, we ask that you include a short, non-technical Author Summary of your research to make findings accessible to a wide audience that includes both scientists and non-scientists. The Author Summary should immediately follow the Abstract in your revised manuscript. This text is subject to editorial change and should be distinct from the scientific abstract. Ideally each sub-heading should contain 2-3 single sentence, concise bullet points containing the most salient points from your study. In the final bullet point of 'What Do These Findings Mean?', please include the main limitations of the study in non-technical language. Please see our author guidelines for more information: https://journals.plos.org/plosmedicine/s/revising-your-manuscript#loc-author-summary.**

We have added an author summary immediately after the abstract.

**> *	Please express the main results with 95% CIs as well as p values. When reporting p values please report as p<0.001 and where higher as the exact p value p=0.002, for example. Throughout, suggest reporting statistical information as follows to improve clarity for the reader "22% (95% CI [13%,28%]; p</=)". Please be sure to define all numerical values at first use.**

All such requirements have been confirmed.

**> *	Please include page numbers and line numbers in the manuscript file. Use continuous line numbers (do not restart the numbering on each page).**

Page numbers and continuous line numbers have been added.

**> *	Please cite the reference numbers in square brackets. Citations should precede punctuation.**

All reference numbers are now being enclosed in square brackets.

FIGURES AND TABLES

**> *	Please provide titles and legends for all figures and tables (including those in Supporting Information files).**

We have ensured all figure and tables have titles and legends.

**> *	Please define all abbreviations used in each figure/table (including those in Supporting Information files).**

All abbreviations in each figure or table have been defined.

**> *	Please consider avoiding the use of red and green in order to make your figure more accessible to those with color blindness.**

The mentioned colors have been removed from the figures.

SUPPLEMENTARY MATERIAL

**> *	Please note that supplementary material will be posted as supplied by the authors. Therefore, please amend it according to the relevant comments outlined here.**

The supplementary material has been revised accordingly.

**> *	Please cite your Supporting Information as outlined here: https://journals.plos.org/plosmedicine/s/supporting-information**

We have cited our supporting information in the link provided.

REFERENCES

**> *	PLOS uses the numbered citation (citation-sequence) method and first six authors, et al.**

We have confirmed the requirements stated.

**> *	Please ensure that journal name abbreviations match those found in the National Center for Biotechnology Information (NCBI) databases (http://www.ncbi.nlm.nih.gov/nlmcatalog/journals), and are appropriately formatted and capitalised.**

We confirm that the journal name abbreviations are in line with those listed in the NCBI database.

**> *	Where website addresses are cited, please include the complete URL and specify the date of access (e.g. [accessed: 12/06/2023]).**

We have confirmed the requirements stated.

**> *	Please also see https://journals.plos.org/plosmedicine/s/submission-guidelines#loc-references for further details on reference formatting.**

We have read and considered the journal guidelines.

STUDY TYPE-SPECIFIC REQUESTS: RCTs

**> *	PLOS Medicine requires that all trials be prospectively registered in one of registries recognized by WHO. Please ensure that study registration details are included in the Methods section.**

We have included the study registration details in the Methods section.

**> *	Please structure the Methods section using the following sub-headings:** **Study design and participants****, Randomization and masking,** **Procedures, Outcomes, Statistical analysis.**

The subheadings in the Methods section have been changed accordingly.

**> *	The primary and secondary outcomes appear to differ between the submitted manuscript and the protocol [and/or trial registry]. Please clarify and explain all discrepancies between the paper and protocol. If the outcomes were not prespecified in the protocol, please define them in the Methods (Outcomes section) as post hoc and explain why they were added. Post-hoc comparisons should be presented as hypothesis generating rather than conclusive.**

As per our comments to the editors, we have added to the text a description of how we changed the primary endpoints, sample size, and the trial registry during the course of the study. We are also submitting the revised protocol and statistical analysis plan as well as the amendment application form and acceptance documents.

On page 16, lines 269–278.

We changed the primary outcome and sample size during the study period because during the peer review of the protocol paper, a reviewer strongly recommended that the original primary endpoint of cephalic version rate after 2 weeks be clinically unimportant and that the clinically important breech presentation at term (which was initially a secondary endpoint) be the primary endpoint. We discussed and carefully considered this recommendation. A power analysis of the case with the breech presentation at term as the primary endpoint showed that the increase in the sample size was well within the feasible range. Thus, we decided to change the primary endpoint and the sample size, and the ethics committee approved this amendment. We also changed the details in the trial registry.

**> *	Please ensure that all prespecified outcomes (primary, secondary, and exploratory) are listed in the Methods/Outcomes section and indicate whether there are outcomes that are not presented in the current report.**

The number of outcomes in the original and revised protocols remains unchanged. All endpoints described in the protocol are reported in the text.

**> *	Please specify the dates (Month Day, Year) during which study enrollment and follow up occurred.**

The start date for recruiting participants has been specified.

On page 9, lines 153–155.

This study was registered in the UMIN Clinical Trials Registry (UMIN000043613) on March 15, 2021, and participant recruitment began on April 1, 2021.

**> *	Please include absolute numbers wherever you report percentages; eg, n/N (%)**

The absolute numbers have been included.

**> *	Please present the safety data for the study including numbers of specific events and whether or not adverse events are thought to be related to treatment. AEs should be reported in the abstract, per CONSORT and CONSORT-Harms.**

We have added the safety data.

**> *	Please complete the CONSORT checklist (https://www.equator-network.org/reporting-guidelines/consort/) and ensure that all components of CONSORT are present in the manuscript, including how randomization was performed, allocation concealment, blinding of intervention, definition of lost to follow-up, power statement. When completing the checklist, please use section and paragraph numbers, rather than page numbers.**

Our manuscript conforms to the CONSORT checklist.

**> *	Please report your abstract according to CONSORT for abstracts, following the PLOS Medicine abstract structure (Background, Methods and Findings, Conclusions) https://www.equator-network.org/reporting-guidelines/consort-abstracts/**

Our abstract is in line with the CONSORT checklist.

**> *	If your trial had to undergo important modifications in response to extenuating circumstances, please complete the CONSERVE-CONSORT checklist and provide in your Supporting Information; (****https://www.equator-network.org/reporting-guidelines/guidelines-for-reporting-trial-protocols-and-completed-trials-modified-due-to-the-covid-19-pandemic-and-other-extenuating-circumstances-the-conserve-2021-statement/). When completing the checklist, please use section and paragraph numbers, rather than page numbers.**

We have confirmed the requirements stated.

**> *	In keeping with our commitment to Open Science, please include the study protocol document and analysis plan (including any amendments) as Supporting Information to be published with the manuscript if accepted.**

A revised protocol and statistical analysis plan will also be submitted along with the revised manuscript.

**> *	Please note that PLOS Medicine** **requires prospective, public registration of a data sharing plan (as part of mandatory clinical trials registration) for all clinical trials that began enrollment on or after January 1, 2019, in accordance with ICMJE requirements.**

We have confirmed the requirements stated.
