# Supplementary material for: Evaluation of postural therapy using lateral position according to fetal back orientation on breech presentation and breech recurrence (BRLT study): An open-label randomized controlled trial
Source: PLoS Med. 2025 Mar 25;22(3):e1004555. doi: 10.1371/journal.pmed.1004555 (PMC11936219; doi:10.1371/journal.pmed.1004555)
Supplement: Attachment — Submitted filename: Reply_to_Comments_auresp_3.docx [file pmed.1004555.s010.docx]

**Our responses to the editors’ and the reviewers’ comments**

We would like to appreciate for inviting us to revise our manuscript again. We have reviewed and addressed all comments.

**Editors:**

Thank you for the opportunity to revise our manuscript again.

**> For full transparency, please make very clear which, when and why protocol changes were made, and add these to the main manuscript text (Methods section).**

We have added the date and reason why the protocol was modified to the Methods section. The reason and content of the modification is specifically explained in the Outcomes section.

On page 9–10, lines 156–157.

The study protocol was amended on July 7, 2022 (within the study period), based on the external reviewer’s opinion. The protocol has been published in a peer-reviewed open-access journal [27].

On page 16, lines 269–277

We changed the primary outcome and sample size during the study period because when the protocol paper was peer-reviewed, a reviewer strongly recommended that the cephalic version rate after 2 weeks (the original primary endpoint) be clinically unimportant and that the clinically important breech presentation at term (the secondary endpoint initially) be the primary endpoint. We discussed and carefully considered this recommendation. A power analysis of the case with the breech presentation at term as the primary endpoint showed that the sample size increase was within the feasible range. Thus, we decided to change the primary endpoint and the sample size, with the approval from the ethics committee. We also changed the details in the trial registry.

**> It appears as though a protocol amendment was made to switch primary and secondary outcomes (approved by the IRB) 16 months into recruitment; please comment on whether this affected the informed consent provided by participants prior to the protocol amendment. How many/what proportion of the participants in the trial had been recruited at the time of the protocol amendment?**

Given that the primary outcome was changed to a more clinically meaningful endpoint, we concluded no disadvantage to the participants and this change would not affect their willingness of participants who had already participated in the trial. The protocol amendment was announced on the Web and in the hospital. The study description document was also revised and made available to 15 participants whose outcomes were tracked at the time. We responded to any inquiries from participants but received few inquiries about the change. No practical changes occurred in the trial conduct because of changes in the primary outcome, and none of the participants wished to withdraw from the trial. As of July 7, 2022, when the protocol was revised, 96 individuals had been recruited into the BRLT study, accounting for 48% of the planned 200.

**> Please note that the raw data as provided contain potentially identifiable information and is unsuitable for publication in its current form. Please provide fully de-identified data underlying the specific results.**

The raw data were resubmitted with the removal of dates and personally identifiable information.

**Reviewer #1** **:**

Thank you for your statistically appropriate opinions. We have revised and reanalyzed the data according to your instruction, as described below.

**> I think the reporting of risks and risk ratios is generally improved, though the wording of the results of cephalic version at 2 weeks is a little awkward, since the outcome is a positive event, but the RR is given for the adverse outcome of the event not happening. Perhaps, if the paper is accepted, the journal's editorial team can help find a suitable form of words here.**

Thank you for your comment. We will accept the decision of the editorial team as to which notation is more appropriate.

**> I applaud the authors' attempt at a CACE analysis, but I am not sure it is done completely correctly. Perhaps it would be better to leave this out.**

We apologize for our inability. In accordance with your opinion, we have removed the Supplementary Appendix about CACE estimate.

**> For the subgroup analysis, I think I may have been misunderstood. The authors have fitted a logistic regression model to estimate the effect of the intervention with adjustment for parity. This is not an unreasonable thing to do, but not quite what I meant. What I was suggesting is fitting a model with an interaction between the intervention and parity.**

**> Let x1 be defined as 1 for those randomised to the intervention and 0 for those randomised to control**

**> Let x2 be defined as 1 for multiparous women and 0 for nulliparous women**

**> Then, let x3 be defined as x1 multiplied by x2. x3 is then said to measure the interaction between the intervention (x1) and parity (x2).**

**> Then, fit a logistic regression model with x1, x2, and x3 as predictors. The paper should report the p-value for the x3 coefficient. If this is significant, it indicates that the intervention effect in multiparous women is different to the intervention effect in nulliparous women.**

We sincerely apologize that we could not understand your intention. We conducted a logistic regression analysis as per your instructions and added it to the Subgroup Analyses subsection.

On page 27, lines 433–438.

In addition, we analyzed whether the intervention effects were the same or different between two subgroups, namely, primiparous and multiparous women. We used a logistic regression model with an interaction between the intervention and parity, and the obtained p-value for the coefficient measuring the interaction between the intervention and parity was 0.13, which did not indicate statistical significance.

**> Finally, I was not convinced by the reasoning for excluding the woman with a planned caesarean from the PP analyses. I accept that the foetal position may be clinically irrelevant if a caesarean is planned, but from the perspective of the trial, the primary outcome at 37 weeks was still measurable, and provides valid information about the effectiveness of the intervention.**

After a careful discussion, we decided not to exclude the past cesarean section from PPS, based your opinion, and we reanalyzed the PPS.

On page 23, lines 378, Table 1.

On page 25, lines 411–413.

Using a predetermined method, the per-protocol analysis revealed that the intervention group had a statistically significant reduction in pelvic position at 37 weeks (9% [8/90] vs. 21% [15/73]); absolute risk difference, −11.7% [95% CI, −22.6% to −0.68%]; relative risk, 0.43 [95% CI, 0.19 to 0.96]; p = 0.020; Table 3).

On page 26, lines 421, Table 3.

**Reviewer #2:**

**> I am much more comfortable with the language the authors use to describe their intervention, which would enable it to be more easily replicated in other tests.**

Thank you for appreciating our work in the manuscript. We appreciated the terminology suggestion provided by you.

**> My co-reviewers have addressed trial management and statistical concerns thoroughly. I will leave it to their judgement as to whether the revisions adequately address their concerns.**

Thank you for addressing the trial management and statistical concerns. We have ensured your comments are correctly addressed by us. Please go through our replies and let us know if any further information is required.

**Reviewer #3:**

**> The authors have responded well on the comments of the reviewers. I recommend publication.**

We appreciate your approval of publishing our paper.

**> I would suggest to publish the raw de-identified data with the paper.**

We have prepared raw deidentified data for the publication.

**General editorial requests:**

We have confirmed and responded to the requirements stated.

**> *	We ask every co-author listed on the manuscript to fill in a contributing author statement, making sure to declare all competing interests. If any of the co-authors have not filled in the statement, we will remind them to do so when the paper is revised. If all statements are not completed in a timely fashion this could hold up the re-review process. If new competing interests are declared later in the revision process, this may also hold up the submission. Should there be a problem getting one of your co-authors to fill in a statement we will be in contact. Please do not add or remove authors without first discussing this with the handling editor. You can see our competing interests policy here: http://journals.plos.org/plosmedicine/s/competing-interests.**

All authors declared no conflicts of interest, as stated in the submission system.

**> *	Please upload any figures associated with your paper as individual TIF or EPS files with 300dpi resolution at resubmission; please read our figure guidelines for more information on our requirements: http://journals.plos.org/plosmedicine/s/figures. While revising your submission, please upload your figure files to the PACE digital diagnostic tool, https://pacev2.apexcovantage.com/. PACE helps ensure that figures meet PLOS requirements. To use PACE, you must first register as a user. Then, login and navigate to the UPLOAD tab, where you will find detailed instructions on how to use the tool. If you encounter any issues or have any questions when using PACE, please email us at PLOSMedicine@plos.org.**

We have complied with the requirements stated.

**> *	Please ensure that the paper adheres to the PLOS Data Availability Policy (see http://journals.plos.org/plosmedicine/s/data-availability), which requires that all data underlying the study's findings be provided in a repository or as Supporting Information. For data residing with a third party, authors are required to provide instructions with contact information (web or email address) for obtaining the data. Please note that a study author cannot be the contact person for the data. PLOS journals do not allow statements supported by "data not shown" or "unpublished results." For such statements, authors must provide supporting data or cite public sources that include it.**

The abovementioned requirements have been followed.

**> *	We expect all researchers with submissions to PLOS in which author-generated code underpins the findings in the manuscript to make all author-generated code available without restrictions upon publication of the work. In cases where code is central to the manuscript, we may require the code to be made available as a condition of publication. Authors are responsible for ensuring that the code is reusable and well documented. Please make any custom code available, either as part of your data deposition or as a supplementary file. Please add a sentence to your data availability statement regarding any code used in the study, e.g. "The code used in the analysis is available from Github [URL] and archived in Zenodo [DOI link]" Please review our guidelines at https://journals.plos.org/plosmedicine/s/materials-software-and-code-sharing and ensure that your code is shared in a way that follows best practice and facilitates reproducibility and reuse. Because Github depositions can**

**be readily changed or deleted, we encourage you to make a permanent DOI'd copy (e.g. in Zenodo) and provide the URL.**

We have adhered to such requirements.

FORMATTING – GENERAL

**> *	Abstract: Please structure your abstract using the PLOS Medicine headings (Background, Methods and Findings, Conclusions). Please combine the Methods and Findings sections into one section.**

The abstract has been revised according to your comment.

**> *	At this stage, we ask that you include a short, non-technical Author Summary of your research to make findings accessible to a wide audience that includes both scientists and non-scientists. The Author Summary should immediately follow the Abstract in your revised manuscript. This text is subject to editorial change and should be distinct from the scientific abstract. Ideally each sub-heading should contain 2-3 single sentence, concise bullet points containing the most salient points from your study. In the final bullet point of 'What Do These Findings Mean?', please include the main limitations of the study in non-technical language. Please see our author guidelines for more information: https://journals.plos.org/plosmedicine/s/revising-your-manuscript#loc-author-summary.**

We have added an author summary immediately after the abstract.

**> *	Please express the main results with 95% CIs as well as p values. When reporting p values please report as p<0.001 and where higher as the exact p value p=0.002, for example. Throughout, suggest reporting statistical information as follows to improve clarity for the reader "22% (95% CI [13%,28%]; p</=)". Please be sure to define all numerical values at first use.**

All such requirements have been followed.

**> *	Please include page numbers and line numbers in the manuscript file. Use continuous line numbers (do not restart the numbering on each page).**

Page numbers and continuous line numbers have been added accordingly.

**> *	Please cite the reference numbers in square brackets. Citations should precede punctuation.**

All reference numbers cited in the text are now enclosed in square brackets.

FIGURES AND TABLES

**> *	Please provide titles and legends for all figures and tables (including those in Supporting Information files).**

We ensured that all figures and tables have titles and legends.

**> *	Please define all abbreviations used in each figure/table (including those in Supporting Information files).**

All abbreviations used in each figure or table have been defined.

**> *	Please consider avoiding the use of red and green in order to make your figure more accessible to those with color blindness.**

We have removed the mentioned colors from the figures.

SUPPLEMENTARY MATERIAL

**> *	Please note that supplementary material will be posted as supplied by the authors. Therefore, please amend it according to the relevant comments outlined here.**

The supplementary material has been revised accordingly.

**>* Please cite your Supporting Information as outlined here: https://journals.plos.org/plosmedicine/s/supporting-information**

We have cited our supporting information in the link provided.

REFERENCES

**> *	PLOS uses the numbered citation (citation-sequence) method and first six authors, et al.**

These requirements have been applied accordingly.

**> *	Please ensure that journal name abbreviations match those found in the National Center for Biotechnology Information (NCBI) databases (http://www.ncbi.nlm.nih.gov/nlmcatalog/journals), and are appropriately formatted and capitalised.**

The journal name abbreviations are in line with those listed in the NCBI database.

**> *	Where website addresses are cited, please include the complete URL and specify the date of access (e.g. [accessed: 12/06/2023]).**

We have complied with these requirements.

**>* Please also see https://journals.plos.org/plosmedicine/s/submission-guidelines#loc-references for further details on reference formatting.**

We have read and considered the journal guidelines.

RCTs

**> *	PLOS Medicine requires that all trials be prospectively registered in one of registries recognized by WHO. Please ensure that study registration details are included in the Methods section.**

We have included the study registration details in the Methods section accordingly.

**> *	Please structure the Methods section using the following sub-headings:** **Study design and participants****, Randomization and masking,** **Procedures, Outcomes, Statistical analysis.**

The subheadings in the Methods section have been revised according to your instruction.

**> *	Please clarify and explain all discrepancies between the paper and protocol in the main manuscript text. If the outcomes were not prespecified in the protocol, please define them in the Methods (Outcomes section) as post hoc and explain why they were added. Post-hoc comparisons should be presented as hypothesis generating rather than conclusive.**

We have added a note about protocol amendment in accordance with the editor’s comments.

On page 9–10, lines 156–157.

The study protocol was amended on July 7, 2022 (within the study period), based on the external reviewer’s opinion. The protocol has been published in a peer-reviewed open-access journal [27].

On page 16, lines 269–277

We changed the primary outcome and sample size during the study period because when the protocol paper was peer-reviewed, a reviewer strongly recommended that the cephalic version rate after 2 weeks (the original primary endpoint) be clinically unimportant and that the clinically important breech presentation at term (the secondary endpoint initially) be the primary endpoint. We discussed and carefully considered this recommendation. A power analysis of the case with the breech presentation at term as the primary endpoint showed that the sample size increase was within the feasible range. Thus, we decided to change the primary endpoint and the sample size, with the approval from the ethics committee. We also changed the details in the trial registry.

**> *	Please ensure that all prespecified outcomes (primary, secondary, and exploratory) are listed in the Methods/Outcomes section and indicate whether there are outcomes that are not presented in the current report.**

The number of outcomes in the original and revised protocols remains unchanged. All endpoints described in the protocol are reported in the text.

**> *	Please specify the dates (Month Day, Year) during which study enrollment and follow up occurred.**

We have specified the start date for participant recruitment.

On page 9, lines 153–155.

This study was registered in the UMIN Clinical Trials Registry (UMIN000043613) on March 15, 2021, and participant recruitment began on April 1, 2021.

**> *	Please include absolute numbers wherever you report percentages; eg, n/N (%)**

We have included the absolute numbers.

**> *	Please present the safety data for the study including numbers of specific events and whether or not adverse events are thought to be related to treatment. AEs should be reported in the abstract, per CONSORT and CONSORT-Harms.**

We have added the safety data.

**> *	Please complete the CONSORT checklist (https://www.equator-network.org/reporting-guidelines/consort/) and ensure that all components of CONSORT are present in the manuscript, including how randomization was performed, allocation concealment, blinding of intervention, definition of lost to follow-up, power statement. When completing the checklist, please use section and paragraph numbers, rather than page numbers.**

Our manuscript conforms to the CONSORT checklist.

**> *	Please report your abstract according to CONSORT for abstracts, following the PLOS Medicine abstract structure (Background, Methods and Findings, Conclusions) https://www.equator-network.org/reporting-guidelines/consort-abstracts/**

Our abstract has been revised according to the CONSORT checklist.

**> *	If your trial had to undergo important modifications in response to extenuating circumstances, please complete the CONSERVE-CONSORT checklist and provide in your Supporting Information; (****https://www.equator-network.org/reporting-guidelines/guidelines-for-reporting-trial-protocols-and-completed-trials-modified-due-to-the-covid-19-pandemic-and-other-extenuating-circumstances-the-conserve-2021-statement/). When completing the checklist, please use section and paragraph numbers, rather than page numbers.**

We have complied with these requirements.

**> *	In keeping with our commitment to Open Science, please include the study protocol document and analysis plan (including any amendments) as Supporting Information to be published with the manuscript if accepted.**

A revised protocol and statistical analysis plan will be submitted along with the revised manuscript.

**> *	Please note that PLOS Medicine** **requires prospective, public registration of a data sharing plan (as part of mandatory clinical trials registration) for all clinical trials that began enrollment on or after January 1, 2019, in accordance with ICMJE requirements.**

We have complied with the requirements stated.
