# Supplementary material for: Evaluation of postural therapy using lateral position according to fetal back orientation on breech presentation and breech recurrence (BRLT study): An open-label randomized controlled trial
Source: PLoS Med. 2025 Mar 25;22(3):e1004555. doi: 10.1371/journal.pmed.1004555 (PMC11936219; doi:10.1371/journal.pmed.1004555)
Supplement: Attachment — Submitted filename: BRLT study_edits.docx [file pmed.1004555.s011.docx]

Evaluation of  postural therapy on breech presentation and breech recurrence (BRLT study): An open-label, randomized controlled trial

Short title: Lateral position management for breech presentation (BRLT study)

Hiroki Shinmura^1*^, Youhei Tsunoda^2^, Takashi Matsushima^1^, Ryuhei Kurashina^1^, Asako Watanabe^1^, Eika Harigane^1^, Nozomi Ouchi^1^, Shunji Suzuki^2^

^1^ Department of Obstetrics and Gynecology, Nippon Medical School Musashikosugi Hospital, Kawasaki, Kanagawa, Japan

^2^ Department of Obstetrics and Gynecology, Nippon Medical School Hospital, Bunkyo, Tokyo, Japan

**Correspondence to:**

Hiroki Shinmura MD, Department of Obstetrics and Gynecology; Nippon Medical School Musashikosugi Hospital, 1-383 Kosugimachi, Nakahara-ku, Kawasaki-shi, Kanagawa, Japan, 211-8533.

E-mail: [h-shimmura@nms.ac.jp](mailto:h-shimmura@nms.ac.jp)

TEL numbers: 044-733-5181

**Abstract**

**Background**

In Japan, the lateral position method is known as a postural therapy for breech presentation wherein the mother lies down in lateral position according to the orientation of the fetal back. Few studies have formally tested lateral position management for breech presentation, and no method exists to prevent breech recurrence after cephalic version. We hypothesized that postural management comprising a combination of opposite side lateral position for breech presentation and same side lateral position after cephalic version might demonstrate a clinically sufficient effect size on breech presentation.

**Methods and findings**

We conducted a stratified, open-label randomized controlled trial at an academic hospital in Kawasaki, Japan. A total of 200 women diagnosed with breech presentation between 28 + 0 and 30 + 0 gestational weeks were randomized to postural management (n = 100) or control (n = 100). The intervention was instruction every 2 weeks on lying in the lateral position on the opposite side of fetal back for breech presentation and on the same side of fetal back after cephalic version. The primary outcome was the rate of fetuses in breech presentation at 37 weeks of gestation. Breech presentation rate at 37 gestational weeks was X% in the intervention group, using the combination of the opposite-side and same-side lateral positions compared with Y% in the control group. However, we found no statistical significance in the intention-to-treat analysis (11% [11/100] vs. 19% [19/100]; absolute risk difference, −8.0% [95% CI, −17.8% to 1.84%]; relative risk, 0.58 [95% CI, 0.29 to 1.15]; p = 0.11). The per-protocol analysis found the same direction of effect but with statistical significance. In the intention-to-treat analysis, the intervention group had a higher cephalic version rate 2 weeks after the instruction (69% [69/100] vs. 54% [54/100]; absolute risk difference, −15.0% [95% CI, −28.3% to −1.67%]; relative risk, 0.67 [95% CI, 0.47 to 0.96]; p = 0.029), and lower breech presentation recurrence rates (2% [2/91] vs. 10% [9/88]; absolute risk difference, −8.0% [95% CI, −15.0% to −1.02%]; relative risk, 0.22 [95% CI, 0.048 to 0.97]; p = 0.031) than the control group. In the intervention group, three participants experienced discomfort and one participant complained of pain in the lateral abdomen; these symptoms resolved spontaneously.

**Conclusions**

For breech presentation at the beginning of the third trimester, providing postural therapy instruction on opposite side lateral positioning for breech presentation and same side lateral positioning after cephalic version was associated with reduced number of breech fetuses at 37 gestational weeks compared with the control group, but this primary endpoint did not reach statistical significance. In our analysis of secondary endpoints, the intervention group showed a significantly higher rate of cephalic version after 2 weeks and lower rate of breech recurrence. The direction of the effect of postural therapy based on fetal back position on breech presentation was promising, and further research to validate this approach, with consideration for unplanned participant crossover, may be warranted.

**Author summary**

**Why was this study done?**

> In Japan, the lateral position method has been practiced as a postural therapy for breech presentation; however, no randomized controlled trials have been conducted to confirm this effect.

> Theoretically, the same side lateral position method, a modification of the opposite side lateral position method previously known in Japan, can prevent breech presentation recurrence after cephalic version; however, to our knowledge, this has never been demonstrated in a study.

**What did the researchers do and find?**

> We conducted a stratified, open-label randomized controlled trial to examine the effect of lateral position management for breech presentation at an academic hospital in Kawasaki, Japan.

> A total of 200 pregnant women whose fetuses were in breech presentation at 28–30 gestational weeks were randomly assigned to the lateral position management (intervention) group (100 participants) or the control group (100 participants), and the intervention group was then instructed to lie on their side according to fetal back, as shown in a separate figure (Fig 1) every 2 weeks.

> At 37 weeks of pregnancy, breech babies in the intervention group were about half of those in the control group, but this result was not statistically significant.

**What do these findings mean?**

> This study demonstrated the potential of a new concept in managing breech presentation.

> Although further study is needed, the opposite side lateral position may potentially reduce breech babies and the same side lateral position may potentially prevent breech presentation recurrence.

**Introduction**

Breech presentation occurs in 3%–4% of all term pregnancies, and vaginal delivery in breech presentation is associated with perinatal morbidity and mortality compared with planned cesarean section [1]. As a large randomized controlled trial published by Hannah et al. (2000), cases of cesarean section for breech presentation have increased worldwide [1-5]. Conversely, a cohort study showed that under the guidance of experienced physicians, vaginal delivery in breech presentation is as safe as cesarean sections, and there is a movement to rethink vaginal delivery in breech presentation [6]. In either case, cephalic version is important because it may avoid the risks of cesarean section and vaginal delivery for breech presentation. External cephalic version is an established treatment method for breech presentation, which is performed after 36 gestational weeks, but it poses problems such as pain and unsuccessful cases [7-10]. Conversely, treatment for breech presentation before 36 weeks has not yet been established. Postural therapies, such as knee–chest position and deep Trendelenburg position, or acupuncture and moxibustion are known treatments that can be performed before 36 weeks; however, evidence to recommend these treatment remains insufficient [11-19].

Wigand (1812) described the lateral position as effective when correcting oblique position to cephalic position [20]. The lateral position on the opposite side of fetal back for breech presentation (Fig 1, opposite side lateral position, A and B), which is a modification of Wigand’s method, has been performed along with the knee–chest position in Japan since 1943, when this report was published by Taoka (1943) [21]. Our previous cohort study demonstrated its possible usefulness; however, the small effect size of the single lateral position instruction remained an issue in the study [23]. Considering the absence of complications observed in the study and a meta-analysis showed that sleeping in lateral position is safe, long-term lateral position performing to compensate for its small effect size was considered acceptable [24]. Currently, no method exists to prevent breech presentation recurrence after cephalic version. Its effectiveness had never been verified, but the lateral position on the same side of fetal back (Fig 1, same side lateral position, C and D) was theoretically a safe method for preventing breech presentation recurrence after cephalic version [24].

**Fig 1. Opposite side lateral position for breech presentation: (A) The right lateral recumbent position is taken if the fetal back is on the mother’s left side; (B) The left lateral recumbent position is taken if the fetal back is on the mother’s right side.**

**Same side lateral position after cephalic version: (C) The right lateral recumbent position is taken if the fetal back is on the mother’s right side; (D) The left lateral recumbent position is taken if the fetal back is on the mother’s left side.**

Hence, we hypothesized that a combination of multiple sessions of the opposite side lateral position instruction every 2 weeks and the same side lateral position method after cephalic version would provide a clinically sufficient effect size on breech presentation. In this study, we conducted the largest randomized controlled trial of postural therapy for breech presentation to determine whether serial instructions of the opposite side lateral or same side lateral positions can reduce term breech presentation cases.

**Methods**

**Study design and participants**

**Trial design and setting**

We performed a stratified, open-label randomized controlled trial with two parallel groups allocated in a 1:1 ratio to evaluate lateral position management for breech presentation, in comparison with the usual prenatal management care at an academic hospital in Kawasaki, Japan.

The study protocol was designed according to the SPIRIT (Standard Protocol Items: Recommendations for Interventional Trials) 2013, and approved by the ethics committee of Nippon Medical School Musashikosugi Hospital on March 9, 2021 (599-2-69) [25]. This study was registered in the UMIN Clinical Trials Registry (UMIN000043613) on March 15, 2021, and participant recruitment began on April 1, 2021. In reporting findings, this study conformed to the CONSORT (Consolidated Standards of Reporting Trials) 2010 statement [26]. The study protocol was amended on July 7, 2022 (within the study period), based on the external reviewer’s opinion. The protocol has been published in a peer-reviewed open-access journal [27].

**Participants**

Notification of the study was made through the hospital website, with no advertising used. Pregnant women who presented for a prenatal checkup at 28 to 30 gestational weeks, where routine ultrasonographic screening was performed, and exhibited a breech presentation were evaluated by their attending obstetricians for eligibility for the trial. We set 28 to 30 gestational weeks as the start of this study to begin the intervention by 32 gestational weeks, when amniotic fluid volume is generally at its maximum and the fetus may rapidly become less rotatable after that point [28].

Inclusion criteria were as follows: gestational age between 28 + 0 and 30 + 0 gestational weeks, pregnant women undergoing prenatal checkups at our hospital, and breech presentation diagnosed by ultrasonography. Exclusion criteria were as follows: less than 20 years old (considering the social reason that the consent of parent or guardian was also required in Japan), scheduled cesarean delivery (included placenta previa, cesarean delivery history, and myomectomy history), scheduled delivery at another hospital, multiple pregnancy, transverse position, any treatment already in place for breech presentation or complications with difficulty in postural management (included cardiovascular disease).

The participants’ eligibility was evaluated on the same day as the diagnosis of breech presentation, and eligible women were recruited to the study by their obstetricians on the same day. We used preprepared documents to prevent variation in recruiters explaining the trial. All participants were provided written informed consent before randomization.

**Randomization and masking**

The allocation tables were computer-generated by a researcher independent of those who conducted the eligibility assessment and recruitment. Random block sizes of 4, 6, and 8 were used. Considering that primiparous women were less likely to rotate their babies than multiparous women, stratified randomization was conducted by parity (primiparous or multiparous), and two allocation lists were used (one for primiparous women and one for multiparous women) [29, 30]. A consecutively numbered allocation list generated by the computer was sealed by security void stickers. If the sticker was removed, it would leave the word “Void” on the list. Moreover, sealing stickers were put on the back of the list to prevent the allocation from being seen through. Then a nurse at the outpatient section of obstetrics performed the randomization by removing the stickers from the list, who was independent of the investigators responsible for enrollment. Group assignment was immediately communicated to the researchers and participants, with no masking. However, the group allocation was masked to the statistical analyst who conducted the analysis.

**Procedures**

Opposite side lateral position in this study was defined as follows: if the fetal back was on the mother’s left side, the mother took the lateral recumbent position with her right side down; if the fetal back was on the mother’s right side, the mother took the lateral recumbent position with her left side down (Fig 1, opposite side lateral position, A and B). Fetal back position was verified by ultrasonography along with estimated fetal weight and amniotic fluid volume at checkup every 2 weeks, and which side the mother should lie on was also updated every 2 weeks. Women assigned to the intervention group were instructed to take the lateral position three times a day for 15 minutes each time for 2 weeks. They were allowed to remain in the lateral position for more than 15 minutes as long as no physical problems occurred, and each 15 minutes was counted as one session. For example, if they could maintain the lateral position for more than 45 minutes on a given day, they were considered to have performed at least three sessions, and that day was regarded as a successful day. Obstetricians who guided the women in the intervention group were pretrained before trial so that they could correctly instruct the women on which side they should sleep on. Opposite side lateral position instruction was given at checkup every 2 weeks until the pelvic position was changed to the head-first position. After the cephalic version, we instructed the intervention group to perform the same side lateral position.

Herein, same side lateral position in this study was defined as follows: if the fetal back was on the mother’s right side, the mother took the lateral recumbent position with her right side down; if the fetal back was on the mother’s left side, the mother took the lateral recumbent position with her left side down (Fig 1, same side lateral position, C and D). The fetal presentation was confirmed at bi-weekly checkups, and the participant continued to be instructed on the opposite side lateral position if in breech presentation, and the same side lateral position if in head-first presentation until delivery. If the fetus was in the transverse position during the trial, the intervention group was instructed as follows: if the fetal head was on the mother’s left side, the mother took the lateral recumbent position with her right side down; if the fetal head was on the mother’s right side, the mother took the lateral recumbent position with her left side down. We did not recommend the knee–chest position and other treatments for breech presentation. Furthermore, participants were instructed to discontinue the lateral position if they felt uncomfortable during the designated posture and to report the incident. We asked them to fill out a daily position record to confirm that they performed the proper posture. Perinatal care other than the intervention was performed according to the 2020 guidelines for obstetrical practice in Japan.

Likewise, the control group received perinatal care based on the 2020 guidelines for obstetrical practice in Japan. Any other treatments for breech presentation before 36 gestational weeks were not recommended. To reassure women in the control group and prevent them from deviating from the protocol, the researchers explained that postural, acupuncture and moxibustion therapies were not recommended because of lack of evidence, and literature suggested that even if the breech presentation was present at the time of allocation, approximately 80% will return to the head-first presentation by 37 gestational weeks. The control group was not prohibited from taking the lateral position, although the proper side was not explained deliberately to avoid mimicking the theoretically correct posture of the intervention group. The control group was also asked to complete a daily position record form until delivery to confirm the type of posture it took.

If the breech presentation persisted at term, all participants were offered the choice of a scheduled C-section, vaginal delivery or external cephalic version. Cesarean section was scheduled at 38 gestational weeks. We informed all participants in advance that they could withdraw from the trial at any time and for any reason. To avoid possible deviations from the study, written instructions were provided in advance for several anticipated questions and inquiries during the study.

**Outcomes**

The primary endpoint was the percentage of breech presentation cases at 37 gestational weeks. The secondary outcomes were cesarean delivery, cesarean delivery for breech presentation as the primary reason, head presentation 2, 4, and 6 weeks later, breech presentation recurrence after cephalic version, and adverse events. The presentation at each time point was confirmed by ultrasonography each time by the investigators in charge of the antenatal checkup that day. The primary and all secondary endpoints, excluding adverse events, were evaluated by comparing the percentage of fetuses with breech presentation between the intervention and control groups. Only breech presentation recurrence was compared between groups converted to cephalic presentation at least once. To evaluate perinatal prognosis in relation to adverse events, we examined participants’ gestational age at delivery, delivery mode, birth weight, Apgar scores at 1 and 5 minutes, umbilical artery pH at birth, bleeding amount during delivery, and perinatal complications were also collected. To prevent missing data, we regularly conducted study progress-sharing sessions wherein researchers shared information about items that could easily be missing.

We changed the primary outcome and sample size during the study period because when the protocol paper was peer-reviewed, a reviewer strongly recommended that the cephalic version rate after 2 weeks (the original primary endpoint) be clinically unimportant and that the clinically important breech presentation at term (the secondary endpoint initially) be the primary endpoint. We discussed and carefully considered this recommendation. A power analysis of the case with the breech presentation at term as the primary endpoint showed that the sample size increase was within the feasible range. Thus, we decided to change the primary endpoint and the sample size, with the approval from the ethics committee. We also changed the details in the trial registry.

**Sample size**

The sample size was calculated on the basis of our prior cohort study on the lateral position for breech presentation and other previous studies. In our prior cohort study, the cephalic conversion rate at term in the intervention group was 94% (16/17) [23]. Hence, we applied the same rate in the intervention group of the current study. In the control group, the previous rate was 77% (30/39). A study dealing with breech presentation at 28–30 weeks, which is the same gestational age as our study, showed a cephalic conversion rate of 75% (non-cephalic position of 21.4% at 28–30 weeks spontaneously decreased to 5.3% after 37 weeks) [31]. In other studies, the cephalic conversion rate was 84% for breech presentation at less than 28 weeks, but in our study, it was estimated to be lower than 84% because this study was conducted after 28 weeks [32, 33]. Therefore, we selected 80% as the cephalic conversion rate for the control group in the current study. A power analysis was performed so that the 14% absolute risk difference (94% v 80%) could be detected with 80% power at an α value of 0.05 (two-sided) in the χ2 test, with 182 as the required sample size. Effect sizes were calculated using G*Power (version 3.1, Faul, Erdfelder, Lang, and Buchner, Düsseldorf, Germany). We set 100 participants per arm, totaling 200 women, considering the missing value of approximately 10%.

**Statistical analysis**

We assessed the outcomes through intention-to-treat and per-protocol analyses according to a predesigned protocol and analysis plan. The primary and secondary outcomes were two-category data, and the intervention and control groups were compared using the χ2 test (Pearson’s test or Fisher’s test). Unadjusted relative risks were calculated as the main result with 95% confidence intervals. We also calculated the absolute risk reductions. Furthermore, we employed Student’s t-test for normally distributed continuous variables, and the Mann-Whitney *U* test for nonnormally distributed ones. Subgroup analyses were separately performed for primiparous participants and multiparous participants. The intention-to-treat analysis included protocol deviations and compared all participants’ results between the two groups. Participants were considered to have successfully performed the lateral position management if they had performed it on the correct side for at least 45 min in at least half of the 2-week period, and whether the patient was able to perform the lateral postural therapy as per protocol was recorded in the categorical variable.

Considering the nature of the lateral position, we could not prohibit the control group from executing lateral position; therefore, many participants in the control group would probabilistically be in the same posture as the intervention group. Already in the design phase of this study, the first randomized controlled trial to examine the lateral position management which has never been tested with a high level of evidence, we considered that the presence of a potential lateral position group in the control group, as described above, would be a barrier to answering the pure question of whether lying on one’s side is really enough to achieve cephalic version. Therefore, a per-protocol analysis that excluded the potential intervention groups within control group was necessary to verify the true efficacy of the lateral position, as distinct from the clinical effectiveness of the intention-to-treat analysis. The per-protocol analysis compared the intervention group, which excluded participants with a success rate of less than 50%, with a control group, which excluded participants with a rate of correct lateral positioning greater than 50% (participants who had performed almost the same therapy as the intervention group). A risk of attrition bias is possible; however, the analysis was conducted strictly according to the protocol as previously decided.

For missing values, we used mean imputation for continuous variables and pairwise deletion for categorical variables and reported the results when used. A P-value <0.05 was considered statistically significant, and two-sided tests were conducted for all analyses. All statistical data were analyzed using IBM SPSS Statistics (version 29.0, IBM Corp.).

**Patient and public involvement**

Patients and the public were not involved in the design, implementation, or dissemination of this study.

**Results**

Between April 2, 2021 and November 18, 2023, 421 pregnant women who presented for a 28–30 weeks obstetric checkup and had a breech presentation on ultrasonography were evaluated for eligibility in person. Of those, 165 women were ineligible, 256 women met the eligibility criteria (Fig 2). Because of refusal to participate, no approach for recruitment or other reasons, 55 women were not included. Of the 201 participants enrolled in this study, one participant has had a history of cesarean section and was excluded before allocation. Ultimately, 200 participants were randomly assigned, 100 to the intervention group and 100 to the control group. Six of the total participants delivered at other hospitals, but information was provided by the other hospitals and the participants whenever possible. Follow-up was conducted until February 6, 2024, when the outcomes of all 200 patients were confirmed. In the intervention group, two were instructed in the opposite side and eight obtained a success rate of less than 50% in performing the lateral position. In the controls, one received the same instruction as the intervention group just before the cephalic version, three confessed to performing the same lateral positioning technique as the intervention group, and 23 had a success rate of greater than 50% in lateral positioning in the same side as the intervention group by the time of the cephalic version. In addition, one of the controls have had a previous cesarean section after allocation, and a scheduled cesarean section was performed at 38 weeks. All 200 participants were included in the intention-to-treat analysis. The per-protocol analysis included 90 participants in the intervention group and 73 in the control group.

**Fig 2.** **Flowchart of participant screening, enrollment, randomization, and inclusion in the analysis.**

Table 1 shows the characteristics of the intervention group, control group and participants excluded from the per-protocol analysis for attrition bias evaluation. The median number of gestational weeks at randomization was 28 weeks for all groups. Incomplete breech was rare in any groups at baseline. Approximately 40% of all participants had undergone fertility treatment to conceive. None of the characteristics showed noticeable differences between the intervention and control groups. Participants excluded from the per-protocol analysis tended to have a higher number of primiparous women (73% vs. 62%) and fewer multiparous women (27% vs. 38%) than controls, but no significant differences were noted in any of the characteristics. None of the groups showed abnormalities in amniotic fluid volume.

**Table 1.** **Baseline Characteristics of Participants.**

**Intention-to-treat analysis**

As the primary outcome in the intention-to-treat analysis, breech presentation occurred at 37 weeks in 11 participants (11% [11/100]) from the intervention group and 19 (19% [19/100]) from the control group; thus, breech presentation occurred in almost a half of those in the intervention group. However, the difference was not statistically significant (absolute risk difference, −8.0% [95% CI, −17.8% to 1.84%]; relative risk, 0.58 [95% CI, 0.29 to 1.15]; p = 0.11; Table 2). For four participants in the intervention group and six in the control group who delivered prematurely, fetal presentation at delivery was considered as the primary outcome. Two weeks after the start of the positioning instruction, breech presentation was converted into cephalic in 69% (69/100) of the participants in the intervention group; this percentage was significantly more than that in the control group (54% [54/100]) (absolute risk difference, −15.0% [95% CI, −28.3% to −1.67%]); relative risk, 0.67 [95% confidence interval 0.47 to 0.99]; p = 0.029). After 4 or 6 weeks of instruction, the cephalic version rate did not significantly differ between such groups. After cephalic version, the intervention significantly prevented breech presentation recurrence more than the control (2% [2/91] vs. 10% [9/88]; absolute risk difference, −8.0% [95% CI, −15.0% to −1.02%]; relative risk, 0.22 [95% CI, 0.048 to 0.97]; p = 0.031). Participants who had non-cephalic presentation at 37 weeks were offered scheduled cesarean section, external cephalic version, or vaginal delivery, and all chose to undergo a scheduled cesarean section. No women wanted to undergo external cephalic version or vaginal delivery, and all who were non-cephalic at 37 weeks underwent a cesarean section at 38 weeks. Moreover, the overall cesarean section rate was significantly lower in the intervention group than in the control group (23% [23/100] vs. 36% [36/100]; absolute risk difference, −13.0% (95% CI, −25.5% to −0.49%); relative risk, 0.64 (95% CI, 0.41 to 0.99); p = 0.044), although this result included one patient with a previous cesarean section.

**Table 2. Primary and Secondary Outcomes in the Intention-to-treat Analysis.**

**Per-protocol analysis**

Using a predetermined method, the per-protocol analysis revealed that the intervention group had a statistically significant reduction in pelvic position at 37 weeks (9% [8/90] vs. 21% [15/73]); absolute risk difference, −11.7% [95% CI, −22.6% to −0.68%]; relative risk, 0.43 [95% CI, 0.19 to 0.96]; p = 0.020; Table 3). Significant differences were also observed in the rate of cephalic presentation at 2 weeks later, total cesarean section rate, cesarean section rate with breech presentation as the indication, and prevention of breech presentation recurrence, but no significant differences were observed in the rate of cephalic version at 4 and 6 weeks later.

**Table 3.** **Primary and Secondary Outcomes in the Per-protocol Analysis.**

**Subgroup analyses**

In the subgroup analyses restricted to multiparous women, the intervention group had a significantly higher rate of cephalic version after 2 weeks (86% [31/36] vs. 50% [19/38]); absolute risk difference, −15.6% [95% CI, −29.1% to −2.20%]; relative risk, 0.28 [95% CI, 0.02 to 0.67]; p = 0.001) and after 6 weeks (97% [35/36] vs. 79% [30/38]; absolute risk difference, −18.3% [95% CI, −32.3% to −4.25%]; relative risk, 0.13 [95% CI, 0.017 to 1.00]; p = 0.029), and a lower rate of cesarean section (6% [2/36] vs. 26% [10/38]); absolute risk difference, −20.8% [95% CI, −36.6% to −4.89%]; relative risk, 0.21 [95% CI, 0.05 to 0.90]; p = 0.015) than the control group (S1 Table). Conversely, the subgroup analyses restricted to primiparous mothers showed no significant differences between the two groups for all outcomes (S2 Table). In addition, we analyzed whether the intervention effects were the same or different between two subgroups, namely, primiparous and multiparous women. We used a logistic regression model with an interaction between the intervention and parity, and the obtained *p*-value for the coefficient measuring the interaction between the intervention and parity was 0.13, which did not indicate statistical significance.

**Adverse events**

As adverse events in the lateral position instruction group, three cases of discomfort and one case of lateral abdominal pain were reported; nonetheless, all were resolved spontaneously, and the perinatal prognosis was good. In the control group, one experienced uterine rupture at delivery, which is a serious outcome. Other variables, namely, gestational age at delivery, birth weight, Apgar score, umbilical artery pH at birth, and blood loss amount, showed no significant differences between the two groups (Table 4).

**Table 4.** **Obstetric Outcomes.**

**Discussion**

We performed the largest randomized controlled trial of postural therapy for breech presentation to the best of our knowledge and revealed that the lateral position management reduced breech presentation at the beginning of third trimester by about 1/2 compared with the control group at 37 gestational weeks, although this difference was not significant in the intention-to-treat analysis. The per-protocol analysis found the same direction of effect with statistical significance. The intention-to-treat analysis further showed significant differences between the two groups in head-first rates 2 weeks after the instruction and breech presentation recurrence rates after cephalic version. These findings are clinically important because they indicate the possibility of reducing breech presentation and preventing breech recurrence just by lying down as long as the fetal back position is known.

**Comparison with other studies**

Currently, no randomized controlled trials have tested the lateral position management for breech presentation, and all randomized controlled trials that have validated postural therapy for breech presentation have been related to the knee–chest position or the deep Trendelenburg position [11-14]. Conversely, one knee–chest position trial showed a higher cephalic version rate in the control group, and a meta-analysis found that the postural management for breech presentation has no sufficient supporting evidence and that further research is needed [11, 12]. In our exploratory cohort study examining the effect of opposite side lateral position, the cephalic version rate at 37 weeks was 94% v 77%, whereas that in the present study was 89% v 81% in the intention-to-treat analysis [23]. Excluding protocol deviations, the per-protocol analysis showed a cephalic version rate of 91% v 79%, similar to that in the prior study. Intervention at earlier weeks is generally considered unnecessary because of the high rate of spontaneous version, and most trials began at around 34 weeks; however, this study showed that interventions by 32 weeks, when the fetus is more likely to rotate than later, may be effective.

This study found four cases of adverse events, all of which were minor and resolved spontaneously. Considering the safety of the lateral position originally reported in a meta-analysis, this study also confirms the safety of providing continuous lateral position instructions [24].

**Implications of findings**

Most common postural managements for breech presentation, such as the knee–chest position or the deep Trendelenburg position, are based on the concept of lifting the pelvis to a higher position to float the fetus out of the pelvis and promote rotation, but holding the pelvis up for a long period of time is difficult. By contrast, the lateral position can be performed for a long time, because it is exactly the position in which a pregnant woman can usually sleep comfortably. In this study, the opposite side lateral position method significantly reduced breech presentation in the third trimester without lifting the pelvis after 2 weeks of instruction. This study is the first to experiment the concept of postural therapy to promote fetal self-rotation without lifting the pelvis, thereby offering a new concept for the treatment of breech presentation.

In the same side lateral position (modification of the opposite side lateral position) performed after cephalic version, gravity is used to stabilize the fetus; however, its effectiveness has never been tested. In the present study, breech presentation recurrences were significantly fewer in the intervention group, indicating for the first time that the same side lateral position can prevent breech presentation. If the same side lateral position is proven effective, it would be inventive because no method to prevent breech presentation recurrence after cephalic version has existed. Early therapeutic intervention is not considered essential because of occasional breech presentation recurrence, but preventing breech recurrence by same side lateral position can make intervention from the early third trimester meaningful.

**Strengths and limitations of study**

The strengths of this study are the stratified randomization by parity, which may have a strong influence on the outcome, the confirmation of the outcomes of all patients, few missing values, and the accurate and objective confirmation of fetal position by ultrasonography at each visit. The lateral position method is also cost-free, can be performed safely anywhere, and has advantages from a medical economics perspective. In this study, ultrasonography was used to ensure objectivity in confirming the presentation and position of fetal back; however, if a skilled person can identify the fetal presentation and fetal back by abdominal palpation (Leopold maneuver), performing lateral position management may be possible in places where ultrasound equipment is not available. Currently, the external cephalic version is an established treatment method for breech presentation, but considering its risks and unsuccessful cases, the option of using the opposite side lateral position method as a premedication before the external cephalic version is considered [10, 34]. Meanwhile, the same side lateral position could be applied as a maintenance therapy after a successful external cephalic version. In addition, rotating the fetus at earlier weeks of gestation and maintaining the cephalic presentation have the advantage of reassuring the mother early on.

The greatest limitation of this study is, as mentioned in the Statistical Analysis of Methods section, the presence of a potential lateral position group that happens to be in the same posture as the intervention group within the control group. Additionally, given that this study was open-label, participants in the control group might have intentionally learned and practiced the lateral positioning method secretly; in fact, three participants in the control group confessed that they had practiced the lateral positioning method. Moreover, the intervention group had 10 (10%) deviants in the intervention group. To address these issues caused by spontaneous and deliberate crossover, we performed a per-protocol analysis and found that lateral position management may be useful. Therefore, the lack of a significant difference in the primary outcome in the intention-to-treat analysis may be explained by the influence of these unplanned crossovers on effect size reduction, and further studies are needed to find a significant difference in the breech presentation at 37 gestational weeks in the intention-to-treat analysis; however, then single-center studies are no longer feasible, and large multicenter studies, preferably with clustering and other measures to eliminate any open-label bias, are needed. Finally, although our intention-to-treat analysis showed a significantly lower cesarean rate in the intervention group, it is necessary to take into account the fact that one prior cesarean section was incidentally included in the control group and that all patients who were in breech presentation at term underwent cesarean section, which is now common in Japan [5].

**Conclusion**

The combination of the opposite side lateral and same side lateral position for breech presentation at 28–30 gestational weeks reduced breech presentation at 37 gestational weeks by approximately 1/2 compared with the control group, although not statistically significant in the intention-to-treat analysis. The per-protocol analysis showed the same tendency of the effectiveness with statistical significance. Regarding the secondary outcomes, the lateral positional group had a considerably high rate of cephalic version after 2 weeks of instruction and a low rate of breech recurrence. The direction of these effects is also promising, and this trial presents a new treatment concept for breech presentation, that is, correcting breech presentation and preventing breech recurrence simply by lying down based on fetal back. Although the application of the lateral position method before and after external cephalic version is assumed, further research considering the impact of unscheduled crossover is warranted.

**Acknowledgments**

We thank all the women who participated and all the staff involved in this study. The following individuals were involved in participant recruitment, ultrasound measurements, positional guidance, and outcome assessment: Ayako Takizawa, Aya Ohno, Honglian Shi, Asako Nagashima, Kei Sagawa, Nobuko Kato, Mayu Yamada, Yuki Kaito, Shingo Ogawa and Go Ichikawa. The following individuals were involved in the concept of this trial: Takehiko Fukami, Ikuno Kawabata, Masahiko Kato and Naofumi Okuda. Risa Shimmura made the allocation lists and sealed them. The authors would like to thank Enago for the English language review. Nobuhiko Taniai was responsible for auditing this trial.

**Author Contributions**

HS and YT conceived the study. SS, HS, YT, and TM were involved in the design of the study. HS invented the same side lateral position. YT, TM, and HS were responsible for data quality. YT and SS performed the analysis and its evaluation. RK, AW, EH and NO played a central role in recruiting, conducting the study, and measuring outcomes. HS wrote the draft. All authors critically revised the manuscript with regard to important intellectual contents, approved the final version, and agreed to the journal’s submission policies.

**Data Availability Statement**

The data that support the findings of this study are available from the corresponding author upon reasonable request.

**Competing Interests**

The authors declare no conflicts of interest.

**References**

1.	Hannah ME, Hannah WJ, Hewson SA, Hodnett ED, Saigal S, Willan AR. Planned caesarean section versus planned vaginal birth for breech presentation at term: a randomised multicentre trial. Term Breech Trial Collaborative Group. Lancet. 2000;356: 1375-1383.

2.	Committee on Obstetric Practice. ACOG committee opinion. Mode of term singleton breech delivery. Int J Gynecol Obstet. 2002;77: 65-6.

3.	Carayol M, Blondel B, Zeitlin J, Breart G, Goffinet F. Changes in the rates of caesarean delivery before labour for breech presentation at term in France: 1972-2003. Eur J Obstet Gynecol Reprod Biol. 2007;132: 20-26.

4.	Hehir MP, O’Connor HD, Kent EM, Fitzpatrick C, Boylan PC, Coulter-Smith S, et al. Changes in vaginal breech delivery rates in a single large metropolitan area. Am J Obstet Gynecol. 2012;206: 498.e1-498.e4.

5.	Sagawa K, Suzuki S, Takeda S, Kinoshita K. Trends in mode of breech delivery in Japan. Hypertens Res Preg. 2021;9: 26-29.

6.	Goffinet F, Carayol M, Foidart JM, Alexander S, Uzan S, Subtil D, et al. Is planned vaginal delivery for breech presentation at term still an option? Results of an observational prospective survey in France and Belgium. Am J Obstet Gynecol. 2006;194: 1002-1011.

7.	Clay LS, Criss K, Jackson UC. External cephalic version. J Nurse Midwifery. 1993;38: 72S-79S.

8.	Coco AS, Silverman SD. External cephalic version. Am Fam Physician. 1998;58: 731-738, 742.

9.	ACOG. External cephalic version: ACOG practice bulletin, number 221. Obstet Gynecol. 2020;135: e203-e212.

10.	Hofmeyr GJ, Kulier R, West HM. External cephalic version for breech presentation at term. Cochrane Database Syst Rev. 2015;2015: CD000083.

11.	Hofmeyr GJ, Kulier R. Cephalic version by postural management for breech presentation. Cochrane Database Syst Rev. 2012;10: CD000051.

12.	Founds SA. Clinical implications from an exploratory study of postural management of breech presentation. J Midwifery Womens Health. 2006;51: 292-296.

13.	Kenfack B, Ateudjieu J, Ymele FF, Tebeu PM, Dohbit JS, Mbu RE. Does the advice to assume the knee-chest position at the 36th to 37th weeks of gestation reduce the incidence of breech presentation at delivery? Clin Mother Child Health. 2012;9: 1-5.

14.	Tafazolifar M, Kordi M, Dadgar S, Esmaily H, Mirteimouri M. The effect of knee-chest position on turning breech to cephalic presentation in pregnant women: randomized clinical trial. Iran J Obst Gynecol Infert. 2019;21: 57-64.

15.	Coyle ME, Smith CA, Peat B. Cephalic version by moxibustion for breech presentation. Cochrane Database Syst Rev. 2012;5: CD003928.

16.	Schlaeger JM, Stoffel CL, Bussell JL, Cai HY, Takayama M, Yajima H, et al. Moxibustion for cephalic version of breech presentation. J Midwifery Womens Health. 2018;63: 309-322.

17.	Bue L, Lauszus FF. Moxibustion did not have an effect in a randomised clinical trial for version of breech position. Dan Med J. 2016;63: A5199.

18.	Miranda-Garcia M, Domingo Gómez C, Molinet-Coll C, Nishishinya B, Allaoui I, Roig MD, et al. Effectiveness and safety of acupuncture and moxibustion in pregnant women with noncephalic presentation: an overview of systematic reviews. Evid Based Complement Alternat Med. 2019;2019: 7036914.

19.	Sananes N, Roth GE, Aissi GA, Meyer N, Bigler A, Bouschbacher JM, et al. Acupuncture version of breech presentation: a randomized sham-controlled single-blinded trial. Eur J Obstet Gynecol Reprod Biol. 2016;204: 24-30.

20.	Wigand JH. Drey den medicinischen Facultäten zu Paris und Berlin zur Prüfung übergebene geburtshülfliche Abhandlungen. III. Von einer neuen und leichten Methode, die Kinder zu wenden und ohne grofse Kunst und Gewalt auf die Welt zu befördern. Hamburg 1812;61-62. (German).

21.	Taoka J. Management for breech presentation in pregnancy [Ninshinchu ni okeru Kotsubani no Shochi]. Sanka Fujinka. 1943;11: 40-45. (Japanese).

22.	Sato J, Kamiariya S, Sato M, Masutori K, Arai S, Akimoto Y, et al. Analysis of spontaneous version and cephalic version by lateral position in our hospital [Tohin ni okeru Kotsubani no Shizenshuseiritsu to Sokugaiho niyoru Kyoseikouka no Kento]. Boseieisei. 1994;35: 207. (Japanese).

23.	Shinmura H, Matsushima T, Okuda N, Watanabe A, Nagashima A, Yamada M, et al. Cephalic version by postural management in the lateral position without the knee-chest position for primiparous breech presentation: a retrospective cohort study. J Obstet Gynaecol Res. 2022;48: 703-708.

24.	Cronin RS, Li M, Thompson JM, Gordon A, Raynes-Greenow CH, Heazell AE, et al. An individual participant data meta-analysis of maternal going-to-sleep position, interactions with fetal vulnerability, and the risk of late stillbirth. EClinicalMedicine. 2019;10: 49-57.

25.	Chan AW, Tetzlaff JM, Altman DG, Laupacis A, Gøtzsche PC, Krleža-Jerić K, et al. SPIRIT 2013 statement: defining standard protocol items for clinical trials. Ann Intern Med. 2013;158: 200-207.

26.	Schulz KF, Altman DG, Moher D, CONSORT Group. CONSORT 2010 statement: updated guidelines for reporting parallel group randomised trials. BMJ. 2010;340: c332.

27.	Shinmura H, Matsushima T, Watanabe A, Shi H, Nagashima A, Takizawa A, et al. Evaluating the effectiveness of lateral postural management for breech presentation: study protocol for a randomized controlled trial (BRLT study). Trials. 2023;24: 360.

28.	Brace RA, Wolf EJ. Normal amniotic fluid volume changes throughout pregnancy. Am J Obstet Gynecol. 1989;161: 382-388.

29.	Levin G, Rottenstreich A, Weill Y, Pollack RN. External cephalic version at term: a 6-year single-operator experience. Birth. 2019;46: 616-622.

30.	Westgren M, Edvall H, Nordström L, Svalenius E, Ranstam J. Spontaneous cephalic version of breech presentation in the last trimester. Br J Obstet Gynaecol. 1985;92: 19-22.

31.	Fox AJS, Chapman MG. Longitudinal ultrasound assessment of fetal presentation: a review of 1010 consecutive cases. Aust N Z J Obstet Gynaecol. 2006;46: 341-344.

32.	Scheer K, Nubar J. Variation of fetal presentation with gestational age. Am J Obstet Gynecol. 1976;125: 269-270.

33.	Hickok DE, Gordon DC, Milberg JA, Williams MA, Daling JR. The frequency of breech presentation by gestational age at birth: a large population-based study. Am J Obstet Gynecol. 1992;166: 851-852.

34.	Cillard L, Verhaeghe C, Spiers A, Madzou S, Descamps P, Legendre G, et al. External cephalic version: predictors for success. J Gynecol Obstet Hum Reprod. 2021;50: 102165.

**Supporting Information Captions**

**Table S1. Primary and Secondary Outcomes in Multiparous Women.**

**Table S2. Primary and Secondary Outcomes in Primiparous Women.**

**Supplementary Tables**

**Table S1. Primary and Secondary Outcomes in Multiparous Women.**

**Table S2. Primary and Secondary Outcomes in Primiparous Women.**

**Figures**

**Fig 1.**

**Fig 2.**

**Figure Legends**

**Fig 1.** Opposite side lateral position for breech presentation: (A) The right lateral recumbent position is taken if the fetal back is on the mother’s left side; (B) The left lateral recumbent position is taken if the fetal back is on the mother’s right side.

Same side lateral position after cephalic version: (C) The right lateral recumbent position is taken if the fetal back is on the mother’s right side; (D) The left lateral recumbent position is taken if the fetal back is on the mother’s left side.

**Fig 2.** Flowchart of participant screening, enrollment, randomization, and inclusion in the analysis.
