# Supplementary material for: Evaluation of postural therapy using lateral position according to fetal back orientation on breech presentation and breech recurrence (BRLT study): An open-label randomized controlled trial
Source: PLoS Med. 2025 Mar 25;22(3):e1004555. doi: 10.1371/journal.pmed.1004555 (PMC11936219; doi:10.1371/journal.pmed.1004555)
Supplement: Attachment — Submitted filename: Reply_to_Comments_third.docx [file pmed.1004555.s012.docx]

**Our responses to the editors’ and the reviewers’ comments**

Thank you all for your generous decision. We have addressed all issues.

**Editors:**

We appreciated for the generous decision and helpful comments.

**> Comments on the abstract are in the attached file.**

Thank you for correcting the abstract. We fully accept the modifications.

**> What do you mean by clinically sufficient? Do you mean clinically relevant?**

That is correct. I apologize for the inadequate wording.

On page 2, lines 30–33.

We hypothesized that postural management comprising a combination of opposite-side lateral position for breech presentation and same-side lateral position after cephalic version might demonstrate a clinically relevant effect size on breech presentation.

**> Please clarify what control is.**

We have accurately described the control group.

On page 3, lines 36–38.

A total of 200 women diagnosed with breech presentation between 28 + 0 and 30 + 0 gestational weeks were randomized to postural management (n = 100) or control (no intervention, n = 100) group.

**> Please rephrase this sentence for clarity.**

We have reworded this statement for clarity.

On page 3, lines 38–40.

The intervention was instruction every 2 weeks on lying in the lateral position on the opposite side of fetal back for breech presentation and on the same side of fetal back for head-first presentation.

**> Please also state here the secondary endpoints that you are reporting.**

We have added secondary endpoints.

On page 3, lines 40–44.

The primary outcome was the rate of fetuses in breech presentation at 37 weeks of gestation, and the secondary outcomes were cesarean delivery, cesarean delivery for breech presentation, head presentation 2, 4, and 6 weeks later, breech presentation recurrence, and adverse events.

**> Please provide exact rates.**

We added specific percentages.

On page 3, lines 44–46.

Breech presentation rate at 37 gestational weeks was 11% in the intervention group, using the combination of the opposite-side and same-side lateral positions, compared with 19% in the control group.

**> Was this analysis pre-sepcified? If yes, please so state. If no, please remove from the abstract.**

The per-protocol set analysis was conducted in a predetermined method, and we have described it as such.

On page 3, lines 49–51.

and, the prespecified per-protocol analysis excluding crossover found the same direction of effect but with statistical significance.

**> Was safety, Aes an endpoint? If so, please phrase accordingly.**

Adverse events were a secondary endpoint. We have restated it for clarity.

On page 4, lines 55–57.

Regarding adverse events in the intervention group, three participants experienced discomfort and one participant complained of pain in the lateral abdomen; these symptoms resolved spontaneously.

**> Please specify the exact rate, and not number if the endpoint is rate.**

We have included specific rates as you indicated.

On page 4, lines 59–62.

For breech presentation at the beginning of the third trimester, providing postural therapy instruction on opposite-side lateral positioning and same-side lateral positioning was associated with 8% reduction of breech fetuses at 37 gestational weeks compared with the control group, but this primary endpoint did not reach statistical significance.

**> Edit correct?**

Thank you for the edit. We found no problem.

On page 4, lines 63–64.

Regarding the secondary endpoints, the intervention group showed a significantly higher rate of cephalic version after 2 weeks and lower rate of breech recurrence.

**> Is this occurred in this trial, you need to mention it in the Methods and Findings section of the abstract.**

We have described specific crossovers in the Methods and Findings section.

On page 3, lines 48–49.

In the control group, 23 participants (23%) unknowingly took the same posture as the intervention group,

**> * Title: Please confirm that your title complies with to PLOS Medicine's style. Your title must be nondeclarative and not a question. It should begin with main concept if possible. "Effect of" should be used only if causality can be inferred, i.e., for an RCT. Please place the study design ("A randomized controlled trial," "A retrospective study," "A modelling study," etc.) in the subtitle (ie, after a colon. We suggest: Evaluation of postural therapy on breech presentation and breech recurrence (BRLT study): An open-label, randomized controlled trial**

We have reviewed the title and modified it according to your suggestion to fit the journal's policy.

On page 1, lines 1–4.

Evaluation of postural therapy using lateral position according to fetal back orientation on breech presentation and breech recurrence (BRLT study): An open-label, randomized controlled trial

**> *Abstract: Please include the clinical trial registry number in the abstract.**

We have appended the clinical trial registry number to the abstract.

On page 4, lines 68–69.

**Trial registration**

UMIN Clinical Trials Registry (UMIN000043613).

**> *Figures: *Figure 1 must be the study flow chart. Please reorganize the figures and figure call-outs in the text accordingly.**

We have corrected all the numbers in the figure accordingly.

Figure 1. Figure 2.

**> *Main text: There are numerous language issues thought the text. Please seek the assistance of a native English language speaker or editor to revise the full manuscript. Please also review your manuscript and edit to ensure compliance with our inclusive language requirements** [**https://journals.plos.org/plosmedicine/s/human-subjects-research#loc-categorization**](https://journals.plos.org/plosmedicine/s/human-subjects-research#loc-categorization)

We had the main text re-edited by the English editing company.

**> *Methods: Please include first and last dates of participant enrolment in the Methods section.**

The first and last dates for participant enrollment have been added.

On page 10, lines 160–161.

The first participant was enrolled in the trial on April 2, 2021 and the last participant on November 18, 2023.

**> Please identify and cite in the Methods the institutional review board(s) that approved the study, separate to the ethics committee.**

Our English translation seemed incorrect. Hence, we have corrected it.

On page 9, lines 155–158.

The study protocol was designed according to the Standard Protocol Items: Recommendations for Interventional Trials (SPIRIT) 2013, with approval of the Nippon Medical School Musashikosugi Hospital Ethics Review Board on March 9, 2021 (approval number: 599-2-69) [25].

**> Please also amend the reference to informed consent. As stated on line 179, the wording “All participants were provided written informed consent before randomization” indicates that the participants were provided with informed consent. Please delete** **‘were’ to indicate that the participants themselves provided the consent.**

Regarding informed consent, we have removed ‘were’ from this sentence as you indicated.

On page 11, lines 186–187.

All participants provided written informed consent before randomization.

**> Please make sure all of the secondary endpoints are described and whether they are all reported. Please signpost any exploratory endpoints or post hoc analyses.**

We confirmed no unreported secondary endpoints, exploratory endpoints, or post hoc analyses.

**> In view of the lack of statistically significant difference in the primary endpoint in this study, the change in study protocol, and the crossover events, please ensure that your representation of the study results and conclusions is appropriately tempered.**

We have reviewed and adjusted our results and conclusion to reflect the factors you mentioned.

**> *Funding: please state the study funders and their role in the study design, execution and analysis.The funding statement should include: specific grant numbers, initials of authors who received each award, URLs to sponsors’ websites. Also, please state whether any sponsors or funders (other than the named authors) played any role in study design, data collection and analysis, the decision to publish, or preparation of the manuscript. If they had no role in the research, include this sentence: “The funders had no role in study design, data collection and analysis, decision to publish, or preparation of the manuscript.**

We have added the funding statement as instructed.

On page 36, lines 594–595.

**Funding**

No funding was received for this study.

**> *Author contributions: Please clarify by what is meant by “HS invented the same side lateral position” . Do you mean that HS invented this approach to management of breech position? Please restate.**

We apologize for the unclear wording.

On page 35, lines 583–584.

HS invented the same side lateral position as an approach to prevent breech presentation recurrence.

**> *Data Availability: Please revise the Data Availability Statement as we do not permit the use of “available upon reasonable request”. For each data source used in your study:**

**> a) If the data are freely or publicly available, note this and state the location of the data: within the paper, in Supporting Information files, or in a public repository (include the DOI or accession number).**

**> b) If the data are owned by a third party but freely available upon request, please note this and state the owner of the data set and contact information for data requests (web or email address). Note that a study author cannot be the contact person for the data.**

**> c) If the data are not freely available, please describe briefly the ethical, legal, or contractual restriction that prevents you from sharing it. Please also include an appropriate contact (web or email address) for inquiries (again, this cannot be a study author).**

The Data Availability Statement was rewritten to accommodate de-identified data submission.

On page 36, lines 590–592.

**Data Availability Statement**

All data supporting the findings of this study are stored in the manuscript and the Supporting Information.

**> * PLOS Medicine requires that the de-identified data underlying the specific results in a published article be made available, without restrictions on access, in a public repository or as Supporting Information at the time of article publication, provided it is legal and ethical to do so. Please see the policy at**

**http://journals.plos.org/plosmedicine/s/data-availability**

**and FAQs at**

**http://journals.plos.org/plosmedicine/s/data-availability#loc-faqs-for-data-policy**

**Please include the deidentified data in the manuscript, as requested by the reviewers.**

We have included de-identified data within the Supporting Information and added the clinical trial registry number to the abstract.

Supporting Information.

De-identified Data set of BRLT study

**> * As your trial had to undergo important modifications in response to extenuating circumstances, please complete the CONSERVE-CONSORT checklist and provide in your Supporting Information.**

**When completing the checklist, please use section and paragraph numbers, rather than page numbers. When completing the CONSORT checklist, please use section and paragraph numbers rather than page numbers.**

In line with your suggestion, we are submitting the CONSERVE-CONSORT checklist and revised the CONSORT checklist.

Supporting Information.

CONSERVE-CONSORT checklist

CONSORT checklist

**> *Protocol: Please provide an English language only protocol.**

We are submitting English language–only protocols.

Supporting Information.

Study Protocol ver1.0

Study Protocol ver2.1

**Reviewer #1** **:**

We thank you for your statistical peer review and helpful opinions. We have revised the paper with additional analysis according to your instruction.

**> Mostly these are fine. The authors have done an interaction test as part of their subgroup analyses, but unfortunately did not state which outcome it related to. I assume it is the primary outcome, but it is not clear. However, this should be done for all outcomes. Currently, the paper highlights certain significant results in the multiparous subgroup, in contrast to no significant results in the primiparous group.**

**> In general, when reporting subgroup analyses, it is not enough to look at the p-values, and to comment on the significance or otherwise of associations within subgroups. Only by testing the interactions do you have evidence of whether the associations are actually different in one subgroup compared to the other.**

**> If possible, I would merge Tables S1 and S2 into a single table (probably in landscape format), and add a column at the end showing the interaction p-values - one for each outcome. This would at least allow the reader to fully assess the results reported.**

We apologize for omitting which outcome the analysis was for. We tested for interactions for all outcomes in the subgroups. Supplemental Tables S1 and S2 were merged, and the p-values obtained in the additional analysis are shown at the far right of the new Supplemental Table.

On page 27, lines 442–446.

We used a logistic regression model to analyze the interaction between the intervention and the parity. Table S presents the obtained p-value of the coefficient measuring the interaction for each outcome. Only the rate of cephalic version after 2 weeks indicated statistical significance (p = 0.014).

Supporting Information.

Table S.
